# Comparative Genomics of Large Mitochondria in Placozoans

**DOI:** 10.1371/journal.pgen.0030013

**Published:** 2007-01-12

**Authors:** Ana Y Signorovitch, Leo W Buss, Stephen L Dellaporta

**Affiliations:** 1 Department of Ecology and Evolutionary Biology, Yale University, New Haven, Connecticut, United States of America; 2 Department of Geology and Geophysics, Yale University, New Haven, Connecticut, United States of America; 3 Department of Molecular, Cellular and Developmental Biology, Yale University, New Haven, Connecticut, United States of America; University of Oxford, United Kingdom

## Abstract

The first sequenced mitochondrial genome of a placozoan, *Trichoplax adhaerens,* challenged the conventional wisdom that a compact mitochondrial genome is a common feature among all animals. Three additional placozoan mitochondrial genomes representing highly divergent clades have been sequenced to determine whether the large *Trichoplax* mtDNA is a shared feature among members of the phylum Placozoa or a uniquely derived condition. All three mitochondrial genomes were found to be very large, 32- to 37-kb, circular molecules, having the typical 12 respiratory chain genes, 24 tRNAs, *rnS,* and *rnL*. They share with the *Trichoplax* mitochondrial genome the absence of *atp8, atp9,* and all ribosomal protein genes, the presence of several *cox1* introns, and a large open reading frame containing an intron group I LAGLIDADG endonuclease domain. The differences in mtDNA size within Placozoa are due to variation in intergenic spacer regions and the presence or absence of long open reading frames of unknown function. Phylogenetic analyses of the 12 respiratory chain genes support the monophyly of Placozoa. The similarities in composition and structure between the three mitochondrial genomes reported here and that of *Trichoplax*'s mtDNA suggest that their uncompacted state is a shared ancestral feature to other nonmetazoans while their gene content is a derived feature shared only among the Metazoa.

## Author Summary

Animals typically have much smaller mitochondrial genomes than do nonanimal single-celled organisms and fungi. Whereas animal mitochondrial genomes are characterized by a tightly packed collection of conserved genes and other functional elements, the larger nonanimal mitochondrial genomes generally contain noncoding regions, such as introns and additional genes not present among animals. It has thus been argued that drastic mitochondrial size reduction occurred deep in evolutionary history, before the emergence of animals. In this study, however, we show that the phylum Placozoa, arguably one of the most ancient animal groups, possesses mitochondrial genomes of intermediate size, smaller than the typical nonanimal genome yet much larger than the mitochondrial genome found in typical animals. As in nonanimals, the increased size of the placozoan mitochondrial genome is due to the presence of additional genes, introns, and long noncoding regions. Although other large animal mitochondrial genomes have been discovered, they have been isolated findings in particular species and none encompassed as large a taxonomic group as the level of phylum. Because large mitochondrial genomes are a shared feature among all placozoans and given this phylum's phylogenetic position in the animal tree, we conclude that, contrary to conventional wisdom, the ancestral animal mitochondria was likely a large, noncompacted molecule.

## Introduction

Comparative mitochondrial genomics is becoming a powerful approach to resolving phylogenetic relationships among distantly related taxa (e.g., [1–7], and reviewed in [8,9]). A problem of particular interest to evolutionary biologists has been the order in which animal phyla comprising the lower Metazoa (cnidarians, ctenophores, placozoans, and sponges) diverged. Since these animal phyla are believed to belong to the earliest diverging branches in the animal tree, learning which characteristics they share with our nonanimal relatives and which traits are unique to animals is essential to understanding metazoan evolution. Recently, sequencing of the entire Trichoplax adhaerens (phylum Placozoa) mitochondrial genome [6] made possible phylogenetic comparisons using all shared mitochondrial coding sequences (12 respiratory chain genes in all) across three lower metazoan phyla—Cnidaria, Placozoa, and Porifera—and two outgroup species, the choanoflagellate Monosiga brevicollis and the chytrid fungus *Monoblepharella*. This revised phylogeny not only provides support for the placement of the lineage leading to the placozoan *Trichoplax* as a basal animal phylum but also raises the possibility that the ancestral animal mitochondrial genome could have actually been a large molecule akin to that of *Trichoplax,* instead of a compact molecule similar to that of all other animals.

*Trichoplax* has one of the smallest animal nuclear genomes [10–12] and yet the largest animal mitochondrial genome [6]. Most animal mitochondrial genomes are small, 15- to 20-kb, circular molecules encoding the typical respiratory chain genes (ATP synthase: *atp6* and *atp8,* apocytochrome b: *cob,* cytochrome oxidase: *cox1, cox2,* and *cox3,* and reduced nicotinamide adenine dinucleotide ubiquinone oxireductase: *nad1–6,* and *nad4L*), 22 tRNA genes, and two rRNA genes. These genes are compactly arranged, sometimes overlapping, and usually lacking intronic and intergenic spacer regions. Although large animal mitochondrial genomes have been discovered [13–16], they are relatively rare and owe their large size to secondary expansions such as duplications, AT-rich regions, and multiple short tandem repeats. In contrast, plant, fungi, and protist mitochondrial genomes are often very large, being up to an order of magnitude larger in size than mtDNA found in the typical animal mitochondria. These nonanimal mtDNA encode many additional proteins—in particular, ribosomal proteins, which are completely absent in animal mitochondria—sometimes extra tRNAs, and they often possess intronic and large intergenic spacer regions. The *Trichoplax* mtDNA structurally resembles an intermediate between the large nonanimal and the compact animal mitochondrial genome. Similar to nonanimals*, Trichoplax* has a large, 43,079–base pair mitochondrial genome and extensive intergenic spacer regions, open reading frames (ORFs), and several introns, but, like all other animals, its genome also lacks ribosomal protein genes.

To understand the ancestral animal condition and the unique features that define Metazoa, the molecular and structural features shared between *Trichoplax* and its relatives must be investigated not only in this particular species but in the entire phylum Placozoa. To this end, we sequenced the mitochondrial genomes of three additional, highly divergent placozoans [17,18], and we here report on our findings. Briefly, all placozoan mtDNA sequenced resembled that of *Trichoplax* in that they possess very large, 32- to 37-kb, circular molecules, an identical set of respiratory chain genes and structural RNAs, as well as an intron group I LAGLIDADG endonuclease domain, conserved intron positions in *cox1,* and large intergenic spacer regions. The differences in genome size among the four placozoan mitochondria can be attributed to length variation in spacer regions and the presence or absence of long open reading frames. Our phylogenetic analyses support the monophyly of Placozoa as well as a basal placement among the Metazoa. Mitochondrial genome size reduction thus likely occurred after the emergence of animals.

## Results

### Mitochondrial Genome Comparisons within Placozoa

**Figure 1:**
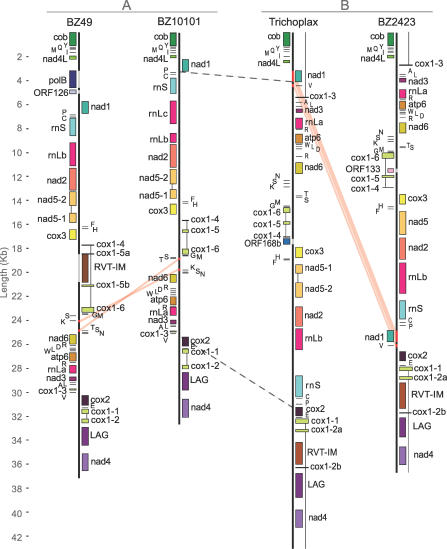
Linearized Scaled Maps of the Four Placozoan Mitochondrial Genomes Each of the four heavy black lines represents the nucleotide sequence of a genome. Genes and RNAs are indicated by their names and color-coded rectangles. Genes transcribed in opposing directions are positioned to the right or left of the sequence line. Introns of *cox1* and *nad5* are denoted by thin black lines connecting each exon. The four mitochondrial genomes were divided into two groups based on their structural and phylogenetic similarity: BZ10101 and BZ49 (A) and *Trichoplax* and BZ2423 (B). Within each group, red and gray lines over the sequence indicate segmental translocations and insertions, respectively. The gray dashed lines across groups (A) and (B) indicate an inversion of the delineated region.

Recent work has shown that Placozoa, once thought to be a monotypic taxon, is actually a phylum composed of no fewer than five highly divergent clades [17,18]. T. adhaerens is a member of Clade I [18]. The complete mitochondrial genome sequences of three placozoan strains: BZ2423, BZ10101, and BZ49, belonging to Clades II, III, and V, respectively [18], were determined and analyzed. They are characteristically large, 32- to 37-kb, circular molecules that contain a common set of 12 respiratory chain genes *(atp6, cob, cox1–3, nad1–6,* and *nad4L),* two ribosomal RNA genes *(rnL* and *rnS),* and a full complement of 24 tRNA genes (having one extra *trnL, trnM, trnR,* and *trnS*). Figure 1 shows the linearized annotated maps of each mitochondrial genome sequenced in this study in addition to that of *Trichoplax* [6]. Many common features were found in all four placozoan strains. *cox1* was found to be distributed across at least six exons on both strands, while the large ribosomal RNA *(rnL)* was split into at least two segments, on the same strand. Another common feature found across all four placozoan mtDNAs was the presence of a large 501– to 677–amino acid ORF containing an intron group I LAGLIDADG endonuclease domain (*LAG*). Its position is conserved between *cox1–2* and *nad4* in all four placozoan mitochondrial genomes.

Each placozoan strain sequenced in this study was unique in its mitochondrial genome content and structure. Table 1 provides a general summary of major features with T. adhaerens included for comparison. Specifically, the largest placozoan mitochondrial genome belongs to *Trichoplax,* at just over 43 kb, followed by BZ2423 (36.7 kb), BZ49 (37.2 kb), and, finally, BZ10101, at 32.7 kb. The percentage of coding plus structural RNA sequences ranged from 55% *(Trichoplax)* to 67% (BZ49), and the median respiratory chain gene length varied from 949.5 bp (BZ10101) to 979.5 bp *(Trichoplax).* BZ10101 and BZ49 had the shortest median intergenic spacer length, at 101 and 105 bp, respectively, while BZ2423 and *Trichoplax* had the longest, at 154 and 209 bp, respectively. BZ10101 presented the lowest G + C content at both genic (coding and RNAs) and nongenic regions—36.4% and 44.4%, respectively, while *Trichoplax* had the highest—39.6% and 56.2%.

**Table 1:**
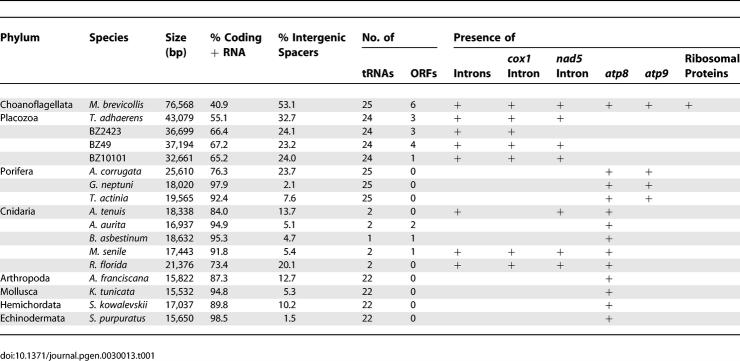
Summary of Mitochondrial Genome Features across Various Metazoa and the Choanoflagellate *Monosiga*

Structural and compositional differences between these four mitochondrial genomes are summarized in Figure 1. A major inversion, depicted by the dotted lines, between *nad1* (or *nad4L*) and *cox2* distinguished BZ10101 and BZ49 (group A) from *Trichoplax* and BZ2423 (group B). Within each group, A or B, two minor segmental inversions or translocations, depicted by the red lines, were also seen. A previously reported large ORF of unknown function containing a reverse transcriptase domain and an intron group II maturase domain *(RVT-IM)* found in *Trichoplax* [6] was also observed in the mtDNA of BZ49 and BZ2423. Unlike *LAG,* the position of *RVT-IM* was not conserved, being found between *cox1-2a* and *cox1-2b* in BZ2423 and *Trichoplax* (group B) and between *cox1-5a* and *cox1-5b* in BZ49 (group A). Amino acid sequence alignment of these *RVT-IM* genes indicated very poor similarity between BZ49 and group B strains (23% to 24% identity, excluding indel sites), while the similarity within group B, excluding indel sites, was 63%. This observation suggested that group A and B *RVT-IM* genes might have originated independently within these placozoans. We also found two positionally conserved exons for *nad5* in the genomes of BZ49, BZ10101, and *Trichoplax* but an intronless *nad5* in BZ2423.

A 491–amino acid ORF similar (BLASTX best match to the fungus, *Hebeloma circinans, E* = 6 × 10^−55^, see Dataset S3 for multiple sequence alignment) to the fungal DNA-directed DNA polymerase type B *(polB)* was identified in the mtDNA of BZ49. To further investigate the origins of this gene, primers designed to conserved regions of *polB* were used to attempt to amplify this gene in BZ10101, BZ2423, and *Trichoplax,* as well as in four additional placozoan isolates belonging to Clade V, to which BZ49 is also a member [18]. The *polB* gene was detected in all members of Clade V but not in other isolates by this method. To determine whether or not the mitochondrial position of *polB* in BZ49 is conserved in this clade, we designed and tested primers targeted at the flanking regions of BZ49 *polB.* Results showed that one other strain, BZ931, contained *polB* in the same position as in BZ49, indicating this gene is located elsewhere in some placozoans from Clade V. This analysis could not distinguish whether the alternate *polB* location or locations were nuclear or mitochondrial.

### Mitochondrial Genome Comparisons and Phylogeny of the Metazoa

Comparisons of certain features across metazoans and the choanoflagellate mitochondria are presented in Table 1. Placozoans have on average the greatest number of ORFs and introns per genome compared to cnidarians, which have up to two introns and ORFs, and poriferans and bilaterians, which have none. The choanoflagellate *Monosiga* has six ORFs and four introns: one in *nad5* and three in *cox1.* Strains BZ49, BZ10101, and *Trichoplax* and all three cnidarians belonging to the subclass Hexacorallia *(Acropora, Metridium,* and *Ricordea),* listed in the table share a conserved intron position with *Monosiga* at *nad5*. Furthermore, *cox1-(1,2,3)* exons in group A animals (BZ49 and BZ10101) and *LAG* in all placozoan strains share conserved positions with *Monosiga*. Placozoan intergenic spacer regions also tended to be greater in number and longer than those of cnidarians, poriferans, and bilaterians. But, like all metazoans, placozoans lack ribosomal proteins*.*

**Figure 2:**
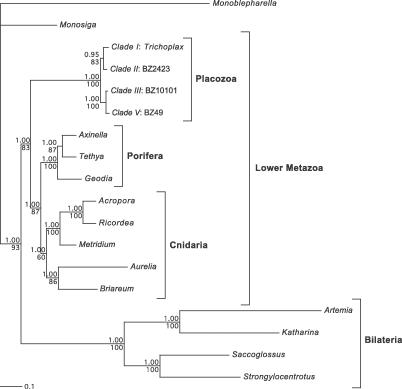
Phylogeny of the Metazoa This phylogenetic tree is based on 2,553 amino acids from 12 concatenated respiratory chain genes (*atp6, cob, cox1–3, nad1–6,* and *nad4L*). Values above internal nodes represent Bayesian posterior probabilities, and those below represent bootstrap percentages under ML. The tree was rooted with the chytrid fungus *Monoblepharella*.

Maximum likelihood (ML) and Bayesian analyses were performed on a dataset containing 12 concatenated protein sequences (*atp6, cob, cox1–3, nad1–6,* and *nad4L*) totaling 2,553 amino acids from 18 taxa (see Dataset S1). Both phylogenetic inference procedures returned the same well-supported tree topology for these taxa (Figure 2). Specifically, the data support a major split in the metazoan tree, with one group containing the lower metazoans and the other containing the bilaterians. These phylogenetic analyses also supported phylum Placozoa as the basal group within the lower Metazoa and, in addition, the two placozoan groups, A and B, that emerged from structural features described.

To determine whether or not the rate of evolution of the placozoan lineage differed significantly from that of the other lower metazoans used in this study, relative-rate tests [19] on each of the 12 respiratory chain genes were performed. Three of the 12 genes produced a significant *p*-value (<0.05), *nad1, nad4,* and *nad5,* indicating that these three genes are likely evolving at different rates between placozoans and the other lower metazoans. However, none of the rates were significantly different after controlling the overall significance level at 5% using the Bonferroni correction.

## Discussion

Sequencing three additional placozoan mtDNAs (BZ49, BZ10101, and BZ2423) allowed us to determine whether the unprecedented features uncovered by the first sequenced placozoan mitochondrial genome [6], that of T. adhaerens, were general phenomena within this phylum. A phylogenetic comparison of these complete placozoan mitochondrial genome sequences to other phyla gave further support for the placement of Placozoa as a basal lower metazoan phylum and provided evidence of the ancestral animal mtDNA condition.

The complete sequences of three additional placozoan mitochondrial genomes revealed that large, noncompact, circular molecules are indeed shared features among members of this phylum. All placozoan mtDNA encoded a common set of 12 respiratory chain genes, 24 tRNAs, two rRNAs, and a large 501– to 677–amino acid ORF containing a group I intron LAGLIDADG endonuclease domain. No *atp8* gene was detected in any of the placozoan mtDNAs sequenced in this study, but because *atp8* is known to be highly variable, we cannot conclusively rule out its presence. Other notable features common to all placozoan mitochondria were the lack of *atp9* and the presence of multiple introns, several unknown ORFs, and relatively large intergenic spacer regions for metazoan genomes. Although their genome size varies considerably (e.g., *Trichoplax* has a mitochondrial genome more than 11 kb larger than that of BZ10101), the gene content, including ORFs of unknown function, across mitochondrial genomes did not show as much variation, ranging between 39 and 42 genes. Even accounting for extra genes, the mitochondrial genome of *Trichoplax* is still larger than any other placozoan mtDNA by at least 3.5 kb. The genome size variation among the placozoan mitochondria was, thus, mainly attributed to differences in intergenic spacer length.

The *cox1* gene of *Trichoplax* was previously found to have an unusual fragmented structure, with exons encoded on different strands of the genome and a large number (at least five) of introns [6]. This fragmentation appears to be a shared feature among all three placozoan strains examined in this study. All genomes contained up to seven *cox1* exons arranged on both strands (Figure 1). Complex gene arrangements have also been observed in the ciliate Tetrahymena pyriformis mitochondrial genome [20], where *nad1* is split into two fragments, one on each strand. Another unusual feature seen in the *Trichoplax* mitochondrial genome, the split in the large subunit ribosomal RNA, *rnL,* into at least two segments on one strand, was also confirmed to be present in other placozoans (Figure 1). Interestingly, the T. pyriformis mitochondrial genome contains a small subunit ribosomal RNA split into two segments [21]. It should be noted, however, that further experimental evidence will be needed to determine whether or not these *rnL* segments in placozoans are spliced together or exist as separate gene segments.

Despite having a common set of respiratory chain genes and structural RNAs, these four placozoan mitochondrial genomes show substantial structural and molecular polymorphisms. While the gene order between BZ49 and BZ10101 and that between BZ2423 and *Trichoplax* were nearly identical, a major inversion between *nad1* (or *nad4L* in BZ2423) and *cox2* distinguished BZ49 and BZ10101 as a separate group from BZ2423 and *Trichoplax*. Molecular phylogenetics based on the 12 respiratory chain genes further supported this division (Figure 2). In addition, the large ORF containing a reverse transcriptase domain and a group II intron maturase domain *(RVT-IM)* was located in the same position in BZ2423 and *Trichoplax* but at a different mitochondrial genomic location in BZ49. The alignment of these genes revealed that the BZ49 *RVT-IM* was highly divergent from the other two (see Dataset S2). Because of this low sequence similarity, the mobile nature of group II introns, and the fact that BZ10101 lacks *RVT-IM* altogether, the origin of *RVT-IM* in BZ49 may have been independent of that of the *RVT-IM* present in BZ2423 and *Trichoplax*.

Phylogenetic analyses were performed using a concatenated amino acid dataset of 12 common respiratory chain genes spanning 18 taxa from the phyla Choanoflagellata, Porifera, Cnidaria, and Placozoa and four bilaterians. A split in the metazoan phylogeny (Figure 2) between two major clades, the Bilateria and lower Metazoa, was observed and may be a result of long-branch attraction due to faster rates of evolution in the Bilateria [6]. Moreover, better resolution of the metazoan phylogeny may be obtained with the eventual addition of other lower metazoan taxa, such as members of the phylum Ctenophora. Nonetheless, this metazoan phylogeny is in agreement with our previous findings [6], supporting Placozoa as a basal lower metazoan phylum. The monophyly of Placozoa was strongly supported by both our comparative mtDNA and phylogenetic analyses. The relatively long branch leading to the phylum Placozoa (Figure 2) additionally suggests an ancient split from other lower metazoans. Relative-rate tests [19] did not show a significant difference in the rate of evolution between the placozoan lineage and the combined sponge and cnidarian lineage compared to the *Monoblepharella* and *Monosiga* outgroups. Furthermore, the branches leading to all placozoan strains are relatively short, indicating that diversification within Placozoa likely occurred in the recent past.

Now that some of the common features among placozoan mtDNA genomes have been described, we can begin to deduce the ancestral condition of the mitochondrial genome of all animals. For example, our data suggest that, contrary to conventional wisdom, the common ancestor of all animals actually possessed large, noncompact mitochondria, owing to the fact that both Placozoa and its closest nonanimal relative, *Monosiga* [22], have distinctively large mitochondrial genomes. Compaction of the mitochondrial genome likely occurred secondarily after the emergence of Metazoa. Our data support the hypothesis that loss of ribosomal protein genes from the mitochondrial genome is a metazoan synapomorphy, as no animal mitochondrial genome sequenced so far, including the large mtDNAs of placozoans described here, has identified sequences coding for ribosomal proteins.

In terms of mitochondrial genome size, structure, and composition, placozoan mitochondrial genomes appear to be intermediates between the very large protist and fungal mtDNAs and the compact animal mtDNAs. Like choanoflagellates and fungi mtDNAs, placozoan mitochondrial genomes have larger protein coding sequences than other lower metazoans and many large intergenic spacers and introns (Table 1). Not only is the *nad5* intron position in placozoans conserved in *Monosiga* and cnidarians, but the positions of the first three *cox1* exons in BZ49 and BZ10101 and the position of *LAG* in all four placozoan genomes are conserved in *Monosiga*. Placozoan mitochondrial genomes share other features similar to most animal mitochondria—predominantly, the lack of all ribosomal protein sequences and the *atp9* gene only found in Porifera [5,23]. Placozoan mtDNAs have some unique features as well. The absence of *atp8* in all four placozoans is likely a synapomorphy for this phylum given its presence in all other taxa sampled in this study (*atp8* is also absent in nematodes and some mollusks; reviewed in [8]). An unusual feature discovered in the BZ49 genome was the presence of a DNA-directed DNA polymerase type B, *polB.* This gene is found in plant and fungal [24] mtDNAs but has only once [25] been previously observed in animal mitochondrial sequences, in the linear mtDNA of the moon jelly Aurelia aurita. However, amino acid sequence alignment of these two animal mitochondrial *polB* genes shows highly diverged sequences, with only 21% identity. Furthermore, the fragmentation and distribution of *cox1* and *rnL* on both placozoans mtDNA strands are features common only to placozoans and no other animal mitochondrial genome sequenced thus far.

Since the Placozoa is a phylum of at least five highly divergent mitochondrial clades [17,18] and our work represents only four of the eight identified 16S rRNA haplotypes, it is likely that by sequencing other mitochondrial genomes we will gain additional insights into both placozoan and metazoan evolution. In particular, with more placozoan taxa sampled, we may be able to determine which taxon belongs to the basal-most placozoan lineage and thus be able to better reconstruct the order of evolutionary events that lead to the diversity of placozoan mitochondrial genomes we observe today. The findings presented here used bioinformatically inferred genes to infer structural and phylogenetic features and, as such, will need experimental confirmation. The resources generated through this study will facilitate experimental studies of placozoan mitochondria. One gene of special interest is *cox1,* which was found arranged on both strands. If *cox1* is experimentally proved to be a viable gene, then the mechanism allowing this protein or proteins to be functional will need to be addressed.

The comparative work presented here demonstrated that animal mitochondrial genomes can be large, not due to repetitive sequences or duplicated regions but due to numerous intergenic spacers, introns, and ORFs of unknown function. The phylum Placozoa, as far as it has been sampled, represents a group of animals with uniquely large mitochondrial genomes. Comparative mitochondrial genomics, both among lower metazoans and within the Placozoa, has proved to be extremely useful in resolving deep phylogenetic relationships. This shared large mitochondrial genome size and basal phylogenetic position among placozoans raises the question of what has allowed the placozoan mitochondrial genomes to remain so large when all other animal mitochondrial genomes have been drastically compacted.

## Materials and Methods

### Placozoan strain selection, cloning, and sequencing.

Three placozoan strains originally isolated from Twin Cays, Belize [18], were chosen for whole mitochondrial genome sequencing: BZ2423, BZ10101, and BZ49. These strains were selected because each represents a different and highly divergent mitochondrial clade [17,18]. Strain BZ2423 belongs to Clade II, BZ10101 belongs to Clade III, and BZ49 belongs to Clade V. Because no formal species description exists for any placozoan species other than *T. adhaerens,* here we referred to the three strains used in our study by their laboratory identification numbers: BZ2423, BZ10101, and BZ49. Each strain was clonally maintained in laboratory cultures, and 2 to 5 μg of total genomic DNA was isolated according to protocols in Signorovitch et al. [26].

Cloning and subcloning of the three placozoan mitochondrial genomes were each carried out as described in the protocols of Dellaporta et al. [6] using pCC1FOS and pSMART LC-Kan (Lucigen, http://www.lucigen.com) vectors, respectively. For each strain, we amplified approximately 384 random subclones by PCR in a 25-μl reaction volume, using the manufacturer's forward and reverse primers, *Taq* polymerase (Qiagen, http://www.qiagen.com), and the following conditions: 95 °C denaturation for 10 min; 30 cycles of 95 °C for 30 s, 57 °C for 30 s, and 72 °C for 4 min; and a 72 °C final extension for 10 min. The amplified products were purified by precipitation using an equal volume of 20% PEG-8000/2.5 M NaCl and then resuspended in 25 μl of 10 mM Tris-Cl (pH 8.0). Purified products were sequenced using PCR primers and TaqFS dye-terminator cycle-sequencing reactions on Prism 3730 DNA sequencers (Applied Biosystems, http://www.appliedbiosystems.com) at the W. M. Keck DNA Sequencing Facility (Yale University) and Genaissance Pharmaceuticals (New Haven, Connecticut, United States).

Certain regions of the mitochondrial genomes of strains BZ10101 and BZ49 contained secondary structures, such as GC-rich hairpin loops, that proved difficult to sequence using the regular methods outlined above. Subclones spanning these regions were selected and plasmid purified from each using the Qiagen Plasmid Purification kit. We used a two-pronged approach to resolving these poor-quality regions. We first attempted sequencing of the purified plasmid using dGTP BigDye polymerase (Applied Biosystems) and the manufacturer's primers. If this first alternative method did not succeed in producing good-quality sequences, we amplified the problematic insert (usually 2 to 4 kb) using Herculase II Fusion (Stratagene, http://www.stratagene.com) polymerase and then sheared the PCR product (approximately 10 μg) by sonication to fragments between 0.5 and 1 kb. The sheared DNA was then end-repaired and cloned into pSMART LC-Kan vector as described above. Approximately 48 random clones were sequenced using the dGTP Big Dye polymerase.

### Sequence analyses.

The DNA sequences of each strain were assembled separately using the Phred, Phrap, and Consed package, release 15.0 [27–29]. Potential genes were identified using the National Center for Biotechnology Information's ORF FINDER, using the Mold, Protozoan, and Coelenterate Mitochondria genetic code. For ORFs of unknown function, only those greater than 100 bp and not overlapping other known genes (i.e., respiratory chain subunits or structural RNAs) were annotated. tRNAs were inferred using the program tRNAscan-SE 1.21 (http://lowelab.ucsc.edu/tRNAscan-SE). Each of the 12 placozoan inferred gene sequences *(atp6, cob, cox1–3, nad1–6,* and *nad4L)* was aligned to its homologous sequence in sponges *(Axinella corrugata*, Geodia neptuni, and *Tethya actinia),* cnidarians *(Metridium senile*, Acropora tenuis, Aurelia aurita, Briareum asbestinum, and *Ricordea florida),* bilaterians *(Artemia franciscana*, Katharina tunicata, Saccoglossus kowalevskii, and *Strongylocentratus purpuratus),* the choanoflagellate *Monosiga brevicollis,* and the chytrid fungus *Monoblepharella* using the program CLUSTALW [30] and edited manually, in order to predict translational start sites and intron-exon boundaries. These alignments were edited using Gblocks, version 0.91b [31], so as to exclude gaps and large nonconserved regions, and the output alignments were concatenated for phylogenetic analyses. Two likelihood-based phylogenetic inference procedures were employed: ML and Bayesian, and both used the mtREV model of amino acid substitution. The program PHYML v2.4.4 [32,33] was used to run the ML analysis and to obtain bootstrap support values (4,000 replicates). The Bayesian analysis was run in MrBayes [34,35], and posterior probabilities were obtained after 500,000 generations with a burn-in of 25%. All parameter values were the same as in Dellaporta et al. [6]. Relative-rate tests were performed on each of the 12 respiratory chain genes individually using the program RRTree [19]. Two lineages were defined: one composed of only placozoan sequences and the other composed of sponge and cnidarian sequences. The outgroup contained *Monoblepharella* and *Monosiga* sequences. A guide tree topology obtained from MrBayes was used in RRTree.

### Amplification of *polB.*

Using strain BZ49 as the reference in designing primer pairs for PCR, we checked for the presence of *polB* in BZ10101, BZ2423, and T. adhaerens [6] and four other Clade V strains (BZ931, BZ322, BZ42, and JM614). Two types of amplification reactions were performed. The first reaction used primers designed to amplify a flanking segment of mitochondrial DNA that included *polB* in BZ49. These reactions used PCR amplification with the use of Herculase II Fusion polymerase and 2% DMSO with the forward 5′-GCTGCAATGGAGGTTGTTTT-3′ and the reverse 5′-ACACCATTTTAAACCCCACCAATC-3′ primer pair and the following PCR conditions: 98 °C for 4 min; 30 cycles at 98 °C for 20 s, 57 °C for 20 s, and 72 °C for 1.5 min; and 72 °C for 3 min. The second reaction was designed to amplify an internal conserved region of *polB* using forward 5′-TCTAAAGATGTTTGATGTGCACTTTT-3′ and the reverse 5′-TTTTGGGCGTTTTTCAACTCTTCT-3′ primer pair and used the same PCR conditions as above.

## Supporting Information

Dataset S1 Concatenated Amino Acid Sequence Data of 12 Respiratory Chain Genes (5.5 MB PDF)

Dataset S2 Amino Acid Sequence Alignment of *RVT-IM*
(68 KB PDF)

Dataset S3 Amino Acid Sequence Alignment of *polB*
(93 KB PDF)

### Accession Numbers

The GenBank (http://www.ncbi.nlm.nih.gov/Genbank) accession numbers for the genes and gene products discussed in this paper are BZ2423, BZ10101, and BZ49, belonging to Clades II, III, and V, respectively (DQ889458, DQ889456, and DQ889457, respectively), A. corrugata (NC_006894), G. neptuni (NC_006990), T. actinia (NC_006991), M. senile (NC_000933), A. tenuis (NC_003522), *A. aurita (*NC_008446), B. asbestinum (NC_008073), R. florida (NC_008159), A. franciscana (NC_001620), K. tunicata (NC_001636), S. kowalevskii (NC_007438), S. purpuratus (X12631), M. brevicollis (NC_004309), and *Monoblepharella* (NC_004624).

## Abbreviations

ML: maximum likelihood
ORF: open reading frame
