# Supplementary material for: Comparative Genomics of Large Mitochondria in Placozoans
Source: PLoS Genet. 2007 Jan 12;3(1):e13. doi: 10.1371/journal.pgen.0030013 (PMC1781491; doi:10.1371/journal.pgen.0030013)
Supplement: Dataset S1 — (5.5 MB PDF) [file pgen.0030013.sd001.pdf]

Dataset S1: Concatenated amino acid sequence data of 12 respiratory chain genes

The following is an amino acid sequence alignment of 12 concatenated respiratory chain genes, whereby the program Gblocks, version 0.91b (Castresana, 2000), has removed all gaps and poorly aligned regions. Characters 1-147 belong to *atp6*, 148-476 to *cob*, 477-936 to *cox1*, 937-1116 to *cox2*, 1117-1355 to *cox3*, 1356-1602 to *nad1*, 1603-1740 to *nad2*, 1741-1805 to *nad3*, 1806-2091 to *nad4*, 2092-2143 to *nad4L*, 2144-2486 to *nad5*, and 2487-2553 correspond to *nad6*.

**Taxon name**	**Abbreviation**
Monoblepharella	PG01
Monosiga	PG02
Trichoplax	PG03
BZ2423	PG04
BZ10101	PG05
BZ49	PG06
Axinella	PG07
Geodia	PG08
Tethya	PG09
Acropora	PG10
Aurelia	PG11
Briareum	PG12
Metridium	PG13
Ricordea	PG14
Artemia	PG15
Katharina	PG16
Saccoglossus	PG17
Strongylocentrotus	PG18

```
PG01 LVPSNWFLGLETYHVTLYTMVRTYIGWFPFLYTLFTGLLFSNLFGLLPYS  50
PG02 LVPSRWQSVMEIIYETVFGVVEDNIGYFPWILTVFMFIVLMNLFGIIPYT  50
PG03 LIPNRVLTGLELIYSHFYTVLKDNLGYLAFVLSLFILILFGNGLGLFPYV  50
PG04 LVPNRILTGLELIYSHFYTVLKDNLGYLAFVLSLFVLILFGNGLGLFPYV  50
PG05 LVPNRILVGFELIFAHFYTVLKDNLGYLAFVLSLFILILFGNGLGLFPYV  50
PG06 LVPNRILVGLELIYTHFYTVLKDNLGYLAFVLSLFILILFGNGLGLFPYV  50
PG07 LIPNRWQSVVELIHLNIRSVVHDNLGYFPFVLSLFIFIAILNILGLFPYV  50
PG08 LIPNRWQFVIEFIYINIHSVVQDNLGFFPFVLCLFIFIAMLNILGLFPYV  50
PG09 LIPNRWQSIMEIIHSNMRSAVHENLGYFPFILCLFLFIAILNILGLFPYV  50
PG10 LIPKRWQSLIELIYEHFHGVVKDNLGYFPLIVSLFFFIVFLNVLGLFPYV  50
PG11 LIPGRWQSIIEIVYDHWIVLVKDSLGYFPFVFSLFLLIAWLNVLGLFPYV  50
PG12 LIPTRLQSIQEIVYTHFLGLVKDSTAYFTLIITLFTFIAGLNLLGLFPYV  50
PG13 LIPNRWQAVMESIYDHFHGLVKDNSGYFPFVFTLFIFIVFLNILGLFPYV  50
PG14 LIPDRWQSIIESIYDHFHGVVKDNLGYLAFIISLFFFIVFLNLLGLFPCV  50
PG15 LIPSRPQFLAKSVLMGLNREMSLLMGANILVIALFLFILFNNFIGLFPYI  50
PG16 FSKSRFYSMILFPKNFMGGQVMRATGFINMVISIFLLLILVNLSGLLPYV  50
PG17 QFPTRFSVLWQTLTNLLPPLFLRSAGWAPLIFALFITLLSNNLLGLTPHS  50
PG18 WAPSRFQSIWLGFRSNILEMIFQNTSWAGLIAGVFVLILLVNVLGLFPAF  50

PG01 TTPTTHLIITFNLALFLMITAIAYRYAIFGLFIPAGIPVALIPVISVVEV  100
PG02 FSPTAHIAVTFGLSLSIFIGVTLHGSNYFSMFMPAGSPLILAPFMIVIEL  100
PG03 FTPTVHMVITLGLSFAIIMGTTLFRLNFFSILMPQGAPLALAPLLTAIET  100
PG04 FTPTVHMVITLGLSFAIIVGTTLFRLNFFSILMPQGAPLALAPLLTAIET  100
PG05 FTPTVHMVITLGLSFAIIVGTTLFRFNFFSILMPQGAPLALAPLLTIIET  100
PG06 FTPTVHMVITLGLSLTIIIGTTLFRFNFFSILMPQGAPLALAPLLTIIET  100
PG07 FTPTAHIVMTFGLSLSILIAVTLFKLNFLSILMPGGVPLMLAPFLVGIET  100
PG08 FVPTAQIVITLGMSVSIIIAVTLFKLNFFSILMPGGVPLVLAPFLVVIET  100
PG09 FTPTAHIIITFGLSLSIMIVVTIFKLDFLSILMPGGIPLALAPFLVIIET  100
PG10 FTPTVHIVVTLGLSFSIIIGVTLFKGDFFSVFMPSGAPLGLAPLLVLIET  100
PG11 FTVGTHIIVTFGLSLSILIIVTIFKADFFSMLMPQGAPMGLAPLLVLIET  100
PG12 FTPTAHIVVTFGLSFSILIAVTIFRWDFASMLMPSGAPLALAPLLVSIET  100
PG13 FTVTVHIVVTLGLSFSIVIGVTLFKWNFLSILMPAGAPLALAPLLVLIET  100
PG14 FTPTVHIVVTLGLSFSIIIGVTLFKWDFFSILMPGGAPLGLAPLLVLIET  100
PG15 FTATSHLAVTLSLAVPLWISFILETTNALAHLVPLGTPAPLMPFMVLMEI  100
PG16 FSLSSHLSFAFCFGLPFWLSLIFYVSEVVSHLLPSGAPGILNPFLVLVET  100
PG17 FTPTSHFSLTLSLAIPLWLAVTINFNLRLSHLLPQGTPPALIPLLVWIEL  100
PG18 QSPTSNISLTYSLGFPLWMAINIAFNSRLSHLVPQGTPSALIPLMVWIET  100

PG01 LAYITRISSLGIRITVNMVTGHTLVKVLELLIAYLQAYIFTFISCITVKR  150
PG02 ISHTAKAVSLGVRLAANITAGHILFAIIEMAVAVIQAYVFSLLTAIYLKT  150
PG03 LSYISRAISLGVRLAANISSGHLLFSILEMAVAIIQAYVFTLLTIVYRLR  150
PG04 LSYISRAISLGVRLAANISSGHLLFSILEMAVAIIQAYVFTLLTIVYRLR  150
PG05 LSYISRAISLGVRLAANISSGHLLFSILEMAVAIIQAYVFTLLTIVYRLR  150
PG06 LSYISRAISLGVRLAANISSGHLLFSILEMAVAIIQAYVFTLLTIVYRLR  150
PG07 LSYMIRAISLGVRLAANISAGHLLFAILEMMVAVIQAYVFCLLTTIYRKE  150
PG08 ASYLIKAISLGVRLAANISAGHLLFAILEIMVAIIQAYVFCLLTTIYRKE  150
PG09 LSYMIRAISLGVRLFANITAGHLLFVILEMAVAIIQAYVFSLLTTIYRKE  150
PG10 VSFISRAISLGVRLAANLSAGHLLFAILEVAVAVIQAYVFCLLATIYRKE  150
PG11 ASYLSRAISLGVRLAANLSAGHLLFAILEIAVAMIQAYVFCLLTTIYRKY  150
PG12 LSYISRAISLGIRLAANLSAGHLLFAILEIAVALIQAYVFCMLTAIYRTQ  150
PG13 VSYISRAISLGVRLAANLSAGHLLFAILEAAVAVIQAYVFSLLTTIYRKE  150
PG14 VSYVSRAISLGIRLAANLSAGHLLFAILEVAVAVIQAYVFCLLTTIYRKV  150
PG15 ISNMIRPITLSVRLAANMIAGHLLLTLLEFSVAIIQSYVFMTLMTLYPEQ  150
PG16 VSISVRPITLSVRLAANMSAGHIILGLFEIGVSLIQAYIFSLLVSLYRSS  150
PG17 LSLLAQPLALALRLAANLTAGHLLIYLLEIAVAVIQAYVFATLTSFYRKT  150
PG18 LSLFAQPIALGLRLAANLTAGHLLIFLLEIGVACIQAYVFTALIHFYRKE  150
```

```
PG01 NPLLALVNDFLIDSPLPANITYFWNTGSLLGVILVIQILTGVILAMHYTP    200
PG02 HPFISIVNNMLIQLPAPSNLSYMWGFGSLLGLCLIIQILTGIFLAMHYCA    200
PG03 KTGLLAPVNDIIDLPTPSNISYWWNFGSLLGLCLGIQILTGVLLAMHYRS    200
PG04 KTGLLAPVNDIIDLPTPSNISYWWNFGSLLSLCLGIQILTGVLLAMHYRS    200
PG05 KTGLLEPVNGIIDLPTPSNISYWWNFGSLLCLCLGIQILTGVLLAMHYRS    200
PG06 KTGLLEPVNDIIDLPTPSNISYWWNFGSLLCLCLGIQIITGVLLAMHYRS    200
PG07 NPVISLINSFLIDLPSPSNISYLWNFGVLLGICLVIQIITGIFLAMHYCS    200
PG08 NPIISLVNNIFVDLPAPSNISYLWNFGSLLGVCLIIQVITGIFLAMHYCA    200
PG09 NPIIVLVNSMFIDLPSPSNISYFWNFGSLLGACLIIQILTGIFLAMHYCS    200
PG10 NPLLSPVNGLLVDLPSPSNISYLWNFGSLLGLCLAMQIVTGCFLSMHYCA    200
PG11 NPLATIINDILVDLPSPSNISYLWNFGSLLGLCLAIQIITGIFLSMHYCS    200
PG12 HPIISIVNGILVDLPAPSNISYLWNFGSLLGACLAIQLITGIFLAMHYCP    200
PG13 NPVLSIVNGIIIDLPAPANLSYMWNFGSLLGVCLVVQMATGIFLAMHYCS    200
PG14 NPLLSLMNSVVVDLPSPSNISYLWNFGSLLGLCLVMQIVTGCFLSMHYCA    200
PG15 QPTLKIINSALVDLPVPANISIWWNFGSLLGLCLLIQIVIGLFLAMHYTA    200
PG16 HPVFKIINGSLIDLPSPSNLSIWWNFGSLLGLCLIIQILTGLFLAMHYAT    200
PG17 NPLISALNSTLVDLPSPSNLSGWWNFGSLLGLCLVSQIITGLFLAMHYTA    200
PG18 HPIFRILNSTFVDLPLPSNLSIWWNSGSLLGLCLVVQILTGIFLAMHYTA    200

PG01 NTLYAFASVEHIDVNYGWLLRYLHANGASFFFIVVYLHIGRGLYFGSYWI    250
PG02 NVDLAFASVEHIDVNAGWLLRYIHANGASMFFIMVYMHIARGLYYGSYWN    250
PG03 DVSLAFSSVAHIDVNYGWILRYVHANGASLFFICVYCHIGRGLYYGSYWI    250
PG04 DVSLAFSSVAHIDVNYGWILRYVHANGASLFFICVYCHIGRGLYYGGYWI    250
PG05 DVSLAFSSVAHIDVNYGWILRYVHANGASLFFICVYCHIGRGLYYGGYWI    250
PG06 DVSLAFSSVAHIDVNYGWILRYVHANGASLFFICVYCHIGRGLYYGGYWI    250
PG07 DVNLAFTSVAHIDVNYGFILRYLHANGASMFFLCVYLHIGRGIYYGGYWN    250
PG08 DVNLAFTSVVHIDVNYGYILRYLHANGASMFFFCVYLHIGRGIYYGSYWN    250
PG09 DVNLAFISVAHIDVNYGFILRYIHANGASLFFLCVYFHIGRGLYYGSYWN    250
PG10 EVGLAFASVGQNDVNYGFLLRYFHANGASLFFLCLYFHIGRSLYYGGYWR    250
PG11 DTTLAFNSVAHIDVNYGFVLKYIHANGASLFFLCVYIHIARGLYYGSYWL    250
PG12 DVNLAFDSVSHIDVNYGFMLKYIHANGASLFFLCVYIHMGRGLYYGSYWN    250
PG13 DVSLAFASVDHIDVNYGFLLRYFHANGASMFFLCLYFHVGRGIYYGSYWN    250
PG14 EVGLAFASVGHIDVNYGFLLRYFHANGASLFFLCLYIHIGRSLYYGGYWG    250
PG15 WVELAFSSVANIDVNYGWLLRTVHANGASFFFICIYFHIGRGMYYGSFWM    250
PG16 NIELAFSSVAHIDVNYGWLIRAAHANGGSLFFICLYFHIGRGMYYGSFWW    250
PG17 DISLAFSSIAHIDVNYGWLLRNLHANGGSLFFICIYLHIGRGLYYGSYWN    250
PG18 DITLAFSSVMHIDVNYGWFLRYVHANGVSLFFICMYCHIGRGLYYGSYWN    250

PG01 VGVFIYIAMMATAFLGYVLPWGQMSFWGATVITNFFSVIPWLGQDIIHFL    300
PG02 IGIIILIVMMATAFIGYVLPWGQMSFWGATVITNFITAIPFIGKDIVEWV    300
PG03 VGVLIYFIMMLTAFIGYVLPWGQMSFWGATVITNLVSAIPSVGGSIVEWI    300
PG04 VGVLIYFIMMLTAFIGYVLPWGQMSFWGATVITNLVSALPSVGGSIVEWI    300
PG05 VGVLIFFIMMLTAFIGYVLPWGQMSFWAATVITNLVSALPSVGSSIVEWI    300
PG06 VGVFIFFIMMLTAFIGYVLPWGQMSFWAATVITNLVSAIPSVGGSIVEWI    300
PG07 VGVLLFFLMILTAFIGYVLPWGQMSFWAATVITNLLSAIPYVGDEIVRWV    300
PG08 VGVLLFLLMIITSFIGYVLPWGQMSFWAATVITNLLSAIPYIGDDIVKWV    300
PG09 VGVILFLLMIITSFIGYVLPWGQMSFWAATVITNLLSAIPYIGDDIVRWV    300
PG10 VGIVIFLLTMATAFLGYVLPWGQMSFWGATVITNLLSAIPYVGTDVVQWV    300
PG11 SGVTIYIIMMATAFIGYVLPWGQMSFWGATVITNLVSAVPYIGNDIVQWL    300
PG12 IGVIIYLIMMLTAFLGYVLPWGQMSFWGATVITNFCSAIPYVGTDIVQWI    300
PG13 VGVVLFILTMATAFLGYVLPWGQMSFWGATVITNLLSAIPYVGTDIVQWV    300
PG14 VGIVIFLLTMATAFMGYVLPWGQMSFWGATVITNLLSAFPYVGTDIVQWV    300
PG15 TGIALLFLVMAAAFLGYVLPWGQMSFWGATVITNLVSAVPYIGNDVVQWL    300
PG16 VGVILLFLTMGTAFIGYVLPWGQMSFWGATVITNLMSAIPYIGTTMVQWI    300
PG17 IGVTLFLATMVTAFLGYVLPWGQMSFWGATVITNLLSAIPCLGQDLVQWV    300
PG18 VGVILFLVTILTAFMGYVLVWGQMSFWAATVITNLVSAIPYIGTIIVQWL    300
```

```
PG01  WGGFSVDHPTLNRFFSFHYLLPFILAALVIIHLLGLHQNGSTNPLSVRGD  350
PG02  WGGFSVSNATLNRFFSLHYLLPFALAGLAVAHLIALHEHGSNNPIGVDSD  350
PG03  WGGFSVSNCTLNRFFSLHYLLPFVLVGLVLAHLLTLHEKGANNPLGVVSD  350
PG04  WGGFSVSNCTLNRFFSLHYLLPFVLVGLVLAHLLTLHEKGSNNPLGVVSD  350
PG05  WGGFSVSNCTLNRFFSLHYLLPFVLVGLVLAHLLTLHEKGSNEPLGLVGD  350
PG06  WGGFSVSNCTLNRFFSLHYLLPFVLVGLVLAHLLTLHEKGSNEPLGLVGD  350
PG07  WGGFSVSNATLNRFFSLHYLLPFVLVGLALVHLIAKHEEGTNNPLGIRSD  350
PG08  WGGFSVSNATLNRFFSLHYLLPFLLIGLALVHLIALHEDGSNNPIGIRSD  350
PG09  WGGFSVSNATLNRFFSLHYLLPFILAGLSLVHLIALHEDGSNNPIGIRSD  350
PG10  WGGFSVSGATLTRFFSLHFLFPFILAILVVVHLIFLHIEGSNSPVGSKTV  350
PG11  WGGFSVSNATLNRFFSLHYLLPFLLAGLGAAHVILLHAAGSSNPVGVKSD  350
PG12  WGGFSVSNATLNRFYSLHYLFPFLIVGLAVLHIISLHTDGSNNPLGISSD  350
PG13  WGGFSVSNATLNRFFSLHYLLPFVLAALGVVHLICLHVSGSNNPIGVRSD  350
PG14  WGGFSVSSATLTRFFSLHFLFPFILAILVGVHLIYLHVEGSNSPIGVKGD  350
PG15  WGGFAVDNPTLTRFFTFHFLIPFLVAGLTMIHLLFLHQSGSNNPLGINAD  350
PG16  WGGFAVDNATLTRFFSFHYLAPFIIAAASVLHILFLHETGSNNPLGINSE  350
PG17  WGGFSINNATLSRFFSFHFILPFIIIVLTLLHLIFLHETGSNNPTGLNSD  350
PG18  WGGFSVDNATLTRFFPFHFLFPFIIAALAVIHLVFLHNSGANNPFAFNSD  350

PG01  YLRFHPYFISKDLVGLGHPDNYIPANPLVTPHHIVPEWYFLPFYAILRAI  400
PG02  KVPFHPYYSVKDVFGLGHSDNYIPANPLVTPQHIVPEWYFLFAYCILRAI  400
PG03  RSTFHVYFTIKDILGLQDPENFIQANPLVTPVHIQPEWYFLFAYAILRSI  400
PG04  RSPFHVYFTIKDILGLQDPENFIQANPLVTPVHIQPEWYFLFAYAILRSI  400
PG05  RTAFHVYFTIKDILGLQDPENFIQANPLVTPVHIQPEWYFLFAYAILRSI  400
PG06  RTAFHVYFTIKDILGLQDPENYIQANPLVTPVHIQPEWYFLFAYAILRSI  400
PG07  MIPFHYYFALKDLYGLSDPENFKQANPLVTPVHIKPEWYFLFAYAILRSI  400
PG08  KVPFHHYYTIKDFFGLGDAENFKQANPLVTPEHIKPEWYFLFAYAILRSI  400
PG09  KVAFHYYYVLKDIYGLGDAENFKPANPLVTPVHIKPEWYFLFAYAILRSI  400
PG10  DVVFHVYYTSKDWYGLGDPENFIQANSLVTPVHIQPEWYFLFAYAILRSI  400
PG11  KIPFHSYFSFKDVYGLGDTENYIQANPLVTPVHIQPEWYFLFAYAILRSI  400
PG12  KVTFHAYYTYKDLFGLGDPENFIQANPLVTPVHIQPEWYFLFAYAILRSI  400
PG13  KVPFHTYYTSKDWYGLGDPENFIQANPLVTPVHIQPEWYFLFAYAILRSI  400
PG14  DVIFHVYYTSKDWYGLGDPENFIQANSLVTPVHIQPEWYFLFAYAILRSI  400
PG15  KLPFHPYFTIKDTVGLGDPDNFIPANPLVTPAHIQPEWYFLFAYAILRSI  400
PG16  KVTIHSFYTLKDLVGLAHPDNFIPANPLVTPTHIQPEWYFLFAYAILRSI  400
PG17  KTSFHSYYSYKDVFGLLDPENFIPANPMSTPVHIQPEWYFLFAYAILRSI  400
PG18  KAPFHIYFTTKDTVGLNDPENFIPANPLVTPPHIQPEWYFLFAYAILRSI  400

PG01  PNKTLGVIAMFGSLLILLALPWFTRLRPMMKVLFWIFVANFFILMWLGGE  450
PG02  PNKLLGVIALFASLVVLAVLPFTHTSKPIAKVFFWFFVADFIILSYLGGQ  450
PG03  PNKLGGVLALFASILVLLLMPILDRSKPVAKFFFWFIVGDFFILTWIGSA  450
PG04  PNKLGGVLALFASILVLLFLPILDRSAPVAKFFFWFIVGDFFILTWIGSA  450
PG05  PNKLGGVLALFASILVLLLMPILDRSKPTGKFFFWFIVGDFIILTWIGSA  450
PG06  PNKLGGVLALFASILVLLLMPILDCSKPTGKFFFWFFVGDFIILTWIGSA  450
PG07  PNKLGGVVALVFSILILLILPYVHINKPLSKILFWFLVGDFILLTWIGGQ  450
PG08  PNKLGGVIALLASILVLFFLPHLHKSRQLSKFFFWFLVGDFFLLTWIGGQ  450
PG09  PNKLGGVVALVFSILVLLVLPLIDQSKPLSKIFFWFLVGDFLLLTWIGGQ  450
PG10  PNKFGGVVSMFLSILILFFFPLLHRSWPFGRMAFWSFVVNFVLLTWIGSL  450
PG11  PDKLGGVIAMAASLLILYALPFISLGMPLAKISYWLFMGNFLLLTWIGSQ  450
PG12  PNKLGGVLAMVASILVLLLLPFIHTSKPLGKLAFWFLIADFILLTWIGAN  450
PG13  PNKLGGVVAMFASLLILFLLPWVHTSRPLARVAFWFMVTDFMLLTWIGCQ  450
PG14  PNKLGGVVSMFFSILILFFLPLLHRSWPFGRLAFWFLVVDFFLLTWIGSQ  450
PG15  PNKLGGVIALVSSILILVSLPFTFKPKSVAQPLFWSWVSVFLLLTWIGAR  450
PG16  PNKLGGVVGLLMSIVILFFVPLINLGKPVTQILYWSLVSVFLLLTWIGAQ  450
PG17  PNKLGGVLALLASVLILLVSPLLHLSKPLSQIFFWTLIANLALLTWIGGQ  450
PG18  PNKLGGVIALVAAILVLFLMPLLNTSKPLSQAAFWLLVAHLFILTWIGSQ  450
```

```
PG01 PIHSPYTEISQVCTFIYFAYFVVLMIRWLFSTNAKDIGLLYIVYAIVGGM 500
PG02 PAEDPYIIIGQLATVFYFSYFIIITPRWVFSTNHKDIGVLYFIFGSFSGF 500
PG03 AGTEPYVLIGRIATIFYFGYFLVLVPRWFFSCNHKDIGSLYLVFGALSGA 500
PG04 AGTEPYVLIGRIATIFYFGYFLVLVPRWFFSCNHKDIGSLYLVFGALSGA 500
PG05 AGADPYVLIGRIATIFYFGYFLVLVPRWFFSCNHKDIGSLYLVFGALSGA 500
PG06 AGADPYVLIGRIATIFYFGYFLVLVPRWFFSCNHKDIGSLYLIFGALSGA 500
PG07 PVEDPYILIGQFAAIFYFAYFLILTPRWLFSTNHKDIGTLYLLLGAFSGM 500
PG08 PVEEPYILIGQLVSLFYFAYFLIIVPRWLYSTNHKDIGTLYLLFGAFSGM 500
PG09 PVEEPYILIGQMASIFYFVYFLILVPRWLFSTNHKDIGTLYLLFGAFAGM 500
PG10 VVEEPFIIIGQLVALYYFFYFLILIPRWGFSTNHKDIGTLYLVFGIGAGM 500
PG11 PVEDPYVLVGQVSTVIYFSYFFLLLPRWLFSTNHKDIGTLYLIFGAFSAM 500
PG12 PVEEPYVLIGQFASMFYFCYFLLLIPRWLFSTNHKDIGTLYLLFGAFSGM 500
PG13 PVEEPFIIVGQLASVFFFAYFLVLSPRWVFSTNHKDIGTLYLVFGIGSGM 500
PG14 VVEEPFIVIGQLVSFFYFSYFFIWVPRWVFSTNHKDIGTLYLVFGVGAGM 500
PG15 PVEDPYNFLGQILTCAYFSYFVFTPIRWFYSTNHKDIGTLYFIFGAWAGM 500
PG16 PVEDPFILIGQILTFVYFLLFLMIPLRWIFSTNHKDIGTLYILFGIWAGL 500
PG17 PVEYPFIEIGQTASSIFFALFVIIIPRWLFSTNHKDIGTLYFIFGAWAGM 500
PG18 PVEYPYVLLGQVASVLYFSLFIFGFPRWLFSTNHKDIGTLYLIFGAWAGM 500

PG01 VGSGISIIMRLELAAPGNLGGNHHLYNVLITAHGLLMIFFMVMPAIIGGF 550
PG02 LGTAMSVIIRMELSIPGSLAGDSHLYNVIVTAHAFLMIFFLVMPFLMGGF 550
PG03 IGTAFSMLIRLELSSPGSMLGDDHLYNVIVTAHAFVMIFFLVMPTMIGGF 550
PG04 IGTAFSMLIRLELSSPGSMLGDDHLYNVIVTAHAFVMIFFLVMPTMIGGF 550
PG05 IGTAFSMLIRLELSSPGSMLGDDHLYNVIVTAHAFVMIFFLVMPTMIGGF 550
PG06 IGTAFSMLIRLELSSPGSMLGDDHLYNVIVTAHAFVMIFFLVMPTMIGGF 550
PG07 IGTAFSVLIRLELSAPGSMLGDDQLYNVIVTAHAFVMIFFLVMPVLIGGF 550
PG08 IGTGFSFLIRLELSAPGSMLGDDHLYNVIITAHGLIMIFFLVMPVMIGGF 550
PG09 IGTGFSMLIRLELSAPGSMLGDDHLYNVIVTAHAFVMIFFLVMPVMIGGF 550
PG10 IGTAFSMLIRLELSAPGAMLGDDHLYNVIVTAHAFIMIFFLVMPVMIGGF 550
PG11 VGTAFSMIIRLELSGPGSMLGDDQLYNVVVTAHALIMIFFFVMPVLIGGF 550
PG12 AGTASSMLIRLELSAPGSMLGDDHLYNVIVTAHALLMIFFLVMPVMIGGF 550
PG13 IGTALSMLIRLELSAPGTMLGDDHLYNVIVTAHAFIMIFFLVMPVMIGGF 550
PG14 IGTAFSVLIRLELSAPGAMLGDDHLYNVIVTAHAFIMIFFLVMPVMIGGF 550
PG15 VGTSLSMLIRAELGQPGSLIGDEQVYNVIVTAHAFIMIFFMVMPILIGGF 550
PG16 VGTALSLLIRAELGQPGALLGDDQLYNVIVTAHAFVMIFFLVMPMMIGGF 550
PG17 IGTGLSLLIRIELAQPGPFLGDDQIYNVIVTAHAFVMIFFMVMPIMIGGF 550
PG18 VGTAMSVIIRAELAQPGSLLNDDQIYNVVVTAHALVMIFFMVMPIMIGGF 550

PG01 GNWLLPIMIGAPDMAFPRLNNLSFWLMPASLTLITLSLLVGNGPGVGWTV 600
PG02 GNFFVPLMIGAPDMSFPRMNNISFWLLPPSLILLVASSLVEGGAGTGWTV 600
PG03 GNWFVPLMIGAPDMAFPRLNNISFWLLPPALFLLLGSSLVEQGAGTGWTV 600
PG04 GNWFVPLMIGAPDMAFPRLNNISFWLLPPALFLLLGSSLVEQGAGTGWTV 600
PG05 GNWFVPLMIGAPDMAFPRLNNISFWLLPPALFLLLGSSLVEQGAGTGWTV 600
PG06 GNWFVPLMIGAPDMAFPRLNNISFWLLPPALFLLLGSSLVEQGAGTGWTV 600
PG07 GNWFVPLYIGAPDMAFPRLNNISFWLLPPSLTLLLGSAFVEQGAGTGWTV 600
PG08 GNWFVPLYIGAPDMAFPRLNNISFWVLPPSLILLLGSAFVEQGVGAGWTV 600
PG09 GNWFVPLYIGAPDMAFPRLNNISFWLLPPSLTLLLGSAFVEQGAGTGWTV 600
PG10 GNWLVPLYIGAPDMAFPRLNNISFWLLPPALILLLGSAFVEQGVGTGWTV 600
PG11 GNWLVPLYIGAPDMAFPRLNNISFWLLPPALLLLLGSSLIEQGAGTGWTI 600
PG12 GNWFVPIMIGAPDMAFPRLNNISFWLLPPSLILLAGSMFVEQGAGTGWTI 600
PG13 GNWLVPLYIGAPDMAFPRLNNISFWLLPPALILLLGSAFVEQGVGTGWTV 600
PG14 GNWLVPLYIGAPDMAFPRLNNISFWLLPPALILLLGSAFVEQGVGTGWTV 600
PG15 GNWLVPIMLGAPDMAFPRLNNLSFWMLPPSLTLLLASSMVESGAGTGWTV 600
PG16 GNWLVPLMLGVPDMAFPRLNNMSFWLLPPALCLLLASGAVESGAGTGWTV 600
PG17 GNWLIPLMLGAPDMAFPRLNNMSFWLLPPAFLLLLSSAAVESGAGTGWTV 600
PG18 GNWLIPLMIGAPDMAFPRMNNMSFWLIPPSFILLLASAGVENGAGTGWTI 600
```

PG01 YPPLSDSLYASGHSVDLAILSLHLAGVSSMVSSINMITTVINMRAPGMTM 650
PG02 YPPLSSVEFHSGGSVDLAIFSLHLAGVSSLLGASNFITTILNMRAPGMTM 650
PG03 YPPLASIQAHSGGSVDMAIFSLHLAGLSSILGAMNFITTVMNMRTPGMTM 650
PG04 YPPLASIQAHSGGSVDMAIFSLHLAGLSSILGAMNFITTVMNMRTPGMTM 650
PG05 YPPLASIQAHSGGSVDMAIFSLHLAGLSSILGAMNFITTVLNMRTPGMTM 650
PG06 YPPLASIQAHSGGSVDMAIFSLHLAGLSSILGAMNFITTILNMRTPGMTM 650
PG07 YPPLASIQAHSGGSVDMVIFSLHLAGISSILGAMNFITTIFNMRAPGITM 650
PG08 YPPLSSVQAHSGGSVDAAIFSLHLAGISSILGSMNFITTIFNMRAPGITM 650
PG09 YPPLASIQAHSGGSVDMAIFSLHLAGISSILSTINFITTILNMRAPGITM 650
PG10 YPPLSSIQAHSGGAVDMAIFSLHLAGVSSILGAMNFITTILNMRAPGMTL 650
PG11 YPPLSSIQAHSGGSVDMAIFSLHLAGASSIMGAINFITTILNMRAPGMTM 650
PG12 YPPLAGIQAHSGGAVDMAIFSLHLAGVSSILSSINFITTIINMRVPGMSM 650
PG13 YPPLSAIQAHSGGAVDMAIFSLHLAGASSILGAMNFITTIFNMRAPGMTM 650
PG14 YPPLSNIQAHSGGAVDMAIFSLHLAGASSILGAMNFITTILNMRAPGITL 650
PG15 YPPLSSAIAHAGPSVDLAIFSLHLAGVSSILGAVNFITTIINMRPQSMSI 650
PG16 YPPLAGNVGHAGGSVDLAIFSLHLAGVSSILGAVNFITTIVNMRSEGMQL 650
PG17 YPPLSGNLAHGGGSVDLAIFSLHLAGISSILGAINFMTTVINMRAAGITW 650
PG18 YPPLSSNITHAGSSVDLAIFSLHLAGASSILGLINFITTIINMRTPGMSL 650

PG01 ERVPLYVWGAFITSFLLVLSLPVLAGGITMLLTDRNFNTTFYDPTGGGDP 700
PG02 HKLPLFVWAVFITAILLLLSLPVLAAGITMLLTDRNFNTSFFDPAGGGDP 700
PG03 SRIPLFVWSVFITAILLLLSLPVLAGAITMLLTDRNFNTTFFDPAGGGDP 700
PG04 SRIPLFVWSVLITAILLLLSLPVLAGAITMLLTDRYFNTTFFDPAGGGDP 700
PG05 SRIPLFVWSVFITAILLLLSLPVLAGAITMLLTDRNFNTTFFDPAGGGDP 700
PG06 SRIPLFVWSVFITAILLLLSLPVLAGAITMLLTDRNFNTTFFDPAGGGDP 700
PG07 DRMPLFVWSILVTVFLLLLSLPVLAGAITMLLTDRNFNTTFFDPAGGGDP 700
PG08 DRLPLFVWSILVTTYLLILSLPVLAGAITMLLTDRNFNTTFFDPAGGGDP 700
PG09 DRMPLFVWSILITAILLLLSLPVLAGAITMLLTDRNFNTAFFDPAGGGDP 700
PG10 NKMPLFVWSILITAFLLLLSLPVLAGAITMLLTDRNFNTTFFDPAGGGDP 700
PG11 DRIPLFVWSVLVTAILLLLSLPVLAGAITMLLTDRNFNTSFFDPAGGGDP 700
PG12 HRLPLFVWSVLITTILLLLSLPVLAGAITMLLTDRNFNTTFFDPAGGGDP 700
PG13 DRLPLFVWSILITAFLLLLSLPVLAGAITMLLTDRNFNTTFFDPAGGGDP 700
PG14 DRMPLFVWSILITAFLLLLSLPVLAGGITMLLTDRNFNTTFFDPAGGGDP 700
PG15 DRMPLFVWAVGITAVLLLLSLPVLAGAITMLLTDRNLNTSFFDPAGGGDP 700
PG16 ERLPLFVWSVKITAILLLLSLPVLAGGITMLLTDRNFNTSFFDPAGGGDP 700
PG17 SRLPLFVWSIFITVILLLLSLPVLAGGITMLLTDRNLNTTFFDPAGGGDP 700
PG18 DRLPLFVWSVFVTAFLLLLSLPVLAGAITMLLTDRNINTTFFDPAGGGDP 700

PG01 VLFQHLFWFFGHPYIMILPGFGIISHVVSRFAVKPVFGSIGMIYAMMSIG 750
PG02 ILYQHLFWFFGHPYILIIPGFGIVSHIVSTFSDKPVFGYLGMVYAMLSIG 750
PG03 ILYQHLFWFFGHYYILILPGFGIISQILSLFSYKQIFGYLGMVKAMLAIG 750
PG04 ILYQHLFWFFGEAYILILPGFGIISQILSLFSYKQIFGYLGMVKAMLAIG 750
PG05 ILYQHLFWFFGKGYILILPGFGIISQILSLFSYKQIFGYLGMVKAMLAIG 750
PG06 ILYQHLFWFFGKGYILILPGFGIISQILSLFSYKQIFGYLGMVKAMLAIG 750
PG07 ILYQHLFWFFGHPYILILPGFGIISQIIPTFAAKQIFGYLGMVYAIVSIG 750
PG08 ILFQHLFWFFGHPYILILPGFGIVSQIIPTFAAKQIFGYLGMVYAMVSIG 750
PG09 ILYQHLFWFFGHPYVLILPGFGIISQIVPTFAAKQIFGYLGMVYAMVSIG 750
PG10 ILFQHLFWFFGHPYILILPGFGMISQIIPTFVAKQIFGYLGMVYAMLSIG 750
PG11 ILFQHLFWFFGHPYILILPGFGIVSQIIPTFSSKQIFGYLGMVYAMIAIG 750
PG12 ILFQHLFWFFGHPYILILPGFGIISQIIPTFSAKHIFGYLGMVYAMISIG 750
PG13 ILFQHLFWFFGHPYILILPGFGMVSQIIPTFSAKQIFGYLGMVYAMLSIG 750
PG14 ILFQHLFWFFGHPYILILPGFGMISQIIPTFVAKQIFGYLGMVYAMLSIG 750
PG15 ILYQHLFWFFGHPYILILPGFGMVSHIISQESGKEAFGTLGMIYAMLAIG 750
PG16 ILYQHLFWFFGHPYILILPGFGMISHIVMHYSSKETFGTLGMIYAMLAIG 750
PG17 ILYQHLFWFFGHPYILILPGFGIISHVIAFYSGKETFGYLGMVYAMLAIG 750
PG18 ILFQHLFWLFGHPYILILPGFGMISHVIAHYSGKEPFGYLGLVYAMIAIG 750

```
PG01  LLGFIVWSHHLYTVGLDVDTRAYFTAATMIIAVPTGIKMFSWLATLYGGI    800
PG02  LLGFIVWAHHMYTVGMDVDTRAYFTASTMIIAVPTGIKIFSWLGTMYGGI    800
PG03  VLGFIVWAHHMFVVGMDVDTRAYFTAATMIIAVPTGIKIFSWLATVSGSL    800
PG04  VLGFIVWADHMYVVGMDVDTRAYFTAATMIIAVPTGIKIFSWLATVSGSL    800
PG05  ILGFIVWADHMYVVGMDVDTRAYFTAATMIIAVPTGIKIFSWLATISGSL    800
PG06  ILGFIVWADHMYVVGMDVDTRAYFTAATMIIAVPTGIKIFSWLATISGSL    800
PG07  ILGFIVWAHHMFTVGMDVDTRAYFTAATMIIAVPTGIKIFSWVATMFGGL    800
PG08  ILGFIVWAHHMFTVGMDVDSRAYFSAATMIIAVPTGIKIFSWIATVVGGL    800
PG09  ILGFIVWAHHMFTVGMDVDTRAYFTAATMIIAVPTGIKIFSWVATMVGGI    800
PG10  ILGFIVWAHHMFTVGMDVDTRAYFTAATMIIAVPTGIKVFSWLATIFGGL    800
PG11  ILGFIVWAHHMFTVGMDVDTRAYFTAATMIIAVPTGIKIFSWLATLYGGV    800
PG12  VLGFIVWAHHMFTVGMDVDTRAYFTAATMIIAVPTGIKIFSWLATIYGGL    800
PG13  ILGFIVWAHHMFTVGMDVDTRAYFTAATMIIAVPTGIKVFSWLATIYGGV    800
PG14  ILGFIVWAHHMFTVDMDVDTRAYFTAATMIIAVPTGIKVFSWLATVYGGL    800
PG15  ILGFVVWAHHMFTVGMDVDTRAYFTAATMIIAIPTGIKIFSWIGTLHGTL    800
PG16  LLGFIVWAHHMFVVGMDVDTRAYFTAATMIIAVPTGIKIFSWLATIYGAI    800
PG17  ILGFLVWAHHMFTVGMDVDTRAYFTAATMVIAVPTGIKIFSWLATLHGSL    800
PG18  VLGFLVWAHHMFTVGMDVDTRAYFTAATMIIAVPTGLKVFSWMAKLQGSL    800

PG01  HLYAPMLFALGFLLLFTMGGVTGVCLANASLDIAFHDTYYVVGHFHYVLS    850
PG02  RLKVPMYWALGFIFLFTLGGITGVMLANGGLDIALHDTYYVVAHFHYVLS    850
PG03  ILTTPMAWALGFIFLFTIGGLTGIVLSNSSLDILLHDTYYVVAHFHFVLS    850
PG04  IFTTPMAWALGFIFLFTIGGLTGIVLSNSSLDILLHDTYYVVAHFHYVLS    850
PG05  IFTTPMAWALGFIFLFTIGGLTGIVLSNSSLDILLHDTYYVVAHFHFVLS    850
PG06  IFTTPMVWALGFIFLFTIGGLTGIVLSNSSLDILLHDTYYVVAHFHFVLS    850
PG07  KLETPMLWAIGFVFLFTLGGLTGIVLANSSLDIVLHDTYYVVAHFHYVLS    850
PG08  RTDTPMLWALGFVFLFTVGGLTGIVLACSSLDVLLHDTYYVVAHFHYVLS    850
PG09  RLDTPMLWAIGFVFLFTIGGLTGIVLANSSLDVVLHDTYYVVAHFHYVLS    850
PG10  RLDTPMLWAMGFVFLFTLGGLTGVVLANSSLDVVLHDTYYVVAHFHYVLS    850
PG11  RFETPMLWAMGFIFLFTLGGLTGIVLANGSLDIALHDTYYVVAHFHYVLS    850
PG12  RLDTPMLWAIGFVFLFTIGGLTGVILANSSLDIMLHDTYYVVAHFHYVLS    850
PG13  RLDTPMLWAIGFVFLFTIGGLTGVILANSSLDIVLHDTYYVVAHFHYVLS    850
PG14  RLDTPMLWALGFVFLFTLGGLTGVVLANSSLDVVLHDTYYVVAHFHYVLS    850
PG15  TMTPSMLWALGFVFLFTVGGLTGVVLSNSSIDIVLHDTYYVVAHFHYVLS    850
PG16  GSETPMMWALGFIFLFTVGGLTGIVLSNSSLDIMLHDSYYVVAHFHYVLS    850
PG17  QWETPLLWALGFVFLFTLGGLTGIVLSNSSLDVVLHDTYYVVAHFHYVLS    850
PG18  QWSLPLLWTLGIVFLFTLGGLTGIVLANSSIDFVLHDTYYVVAHFHYVLS    850

PG01  MGAVFAIFAAFYYWVGKMTGYNYPEYLARVHFWTFFIAVNLVFFPMHFLG    900
PG02  MGAVFALIGGVYYWIGKVTGYAYPETWGKIHFWLMFIGVNLTFFPQHFLG    900
PG03  MGAVFAIFGGFYLWFGKITGYAYKDIYGLIHFWLMFIGVNITFFPQHFLG    900
PG04  MGAVFAIFGGFYLWFGKITGYAYKDIYGLIHFWLMFLGVNITFFPQHFLG    900
PG05  MGAVFAIFGGFYLWFGKITGYAYKDIYGLIHFWLMFVGVNITFFPQHFLG    900
PG06  MGAVFAIFGGFYLWFGKITGYAYKDIYGLIHFWLMFFGVNITFFPQHFLG    900
PG07  MGAVFAMFGGFYYWFGKISGYCYNEVYGKIHFWLMFIGVNLTFFPQHFLG    900
PG08  MGAVFALFGGFYYWFGKITGYCYNEVLGKIHFWLMFIGVNITFFPQHFLG    900
PG09  MGAVFAIFGGFYYWFGKITGYCYNEVYGKIHFWLMFIGVNLTFFPQHFLG    900
PG10  MGAVFAIFGGFYYWVGKITGYCYNELYGKAHFWLMFIGVNLTFFPQHFLG    900
PG11  LGAVFAIFGGFYYWFGKITGYSYNEVYGKIHFWIMFIGVNATFFPQHFLG    900
PG12  MGAVFAIFGGYYYWFGKITGFSYNELYGKIHFWIMFIGVNLTFFPQHFLG    900
PG13  MGAIFAIFAGFYFWFGKITGYCYNELYGKIHFWIMFIGVNLTFFPQHFLG    900
PG14  MGAVFAIFGGFYYWVGKITGYCYNEFYGKVHFWLMFVGVNLTFFPQHFLG    900
PG15  MGAVFAIMAGFVHWFPLMTGLSMNQFLLKVHFFIMFLGVNLTFFPQHFLG    900
PG16  MGAVFALFGAFNYWYPLMTGLSLHERWTKSHFVVMFLGVNLTFFPQHFLG    900
PG17  MGAVFAIFAGFIHWFPLFTGYGLHPVWTKVHFWTMFLGVNVTFFPQHFLG    900
PG18  MGAVFAIFAGFTHWFPLFSGYSLHPLWGKVHFFIMFVGVNLTFFPQHFLG    900
```

```
PG01 INGMPRRIPDYPEAFANWNVVMSLGTVFTVFSLIVFNLSFQDVASPTMYG  950
PG02 LAGFPRRYNDFPDAYAEWNLLSSFGSLISVVAVIVFQLSFQDPASPVFEE  950
PG03 LAGLPRRYADYPDNFEDFNQISSFGSVISLTAVIWFQLGFQDAGSPVMEE  950
PG04 LAGLPRRYADYPDNFEDFNQISSFGSIISIIAVIWFQLGFQDAGSPVMEE  950
PG05 LAGLPRRYADYPDNFEDFNQISSFGSIISIIAVIWFQLGFQDAGSPVMEE  950
PG06 LAGLPRRYADYPDNFEDFNQISSFGSIISIIAVIWFQLGFQDAGSPVMEE  950
PG07 LAGLPRRYSDFHDTFAGWNQISSLGSMISIIGVVWFQIGFQDAASPVMEE  950
PG08 LAGLPRRYSDYHDSFAGWNQISSVGSLISIVSVMVFQLGFQDAASPVMEE  950
PG09 LAGLPRRYSDFHDSFAGWNQISSLGSVISIVGVIWFQLGFQDAASPVMEE  950
PG10 LTGFPRRYSDFADCFAGWNLVSSLGSTISIVGVVFFHLGFQEAAAPVMEE  950
PG11 LAGMPRRYSDFPDSFTGWNIVSSLGSAISIVGVLWFQLSFQDSVSPIMEE  950
PG12 LAGLPRRYSDFADAYQDWNLVSSCGAVIALIGIMWFQLSFQDAASPVMEE  950
PG13 LAGFPRRYSDFVDGFAGWNLVSSLGSTISIVGVIWFQLGLQDAAHPVMEE  950
PG14 LTGFPRRYSDFADSFAGWNLVSSLGSAVVLVGVVWFHLGLQDAADPVMEE  950
PG15 LAGMPRRYADYPDTYASWNVISSLGSVMSMIATLMFQLGLQNGNSPLMEQ  950
PG16 LSGMPRRYSDYPDCYIKWNVVSSIGSLISFVAVLFFQWGFQDGASPIMEQ  950
PG17 LAGMPRRYSDFPDAYTTWNVVSSVGSIISLTSVILFQISLQDAASPIMEE  950
PG18 LAGMPRRYSDYPDAYTLWNTISSIGSTISVVAMLFFQFGLQDASSPLMEE  950
```

```
PG01 LLELHDHVLFYLILILITVGHVIEFLWTLAPALILGAIALPSFRLLYLMD  1000
PG02 IINFHNYVFVYMTFIFFGVGTLIELIWTVTPALVLVGIAFPSFKLLYLMD  1000
PG03 IIMLHDQILFILTIIVGVVGSLIEVIWTLIPAFILILIAFPSLRLLYIMD  1000
PG04 IIMLHDQILFILTIIVGVVGSLIEVIWTLIPAFILILIAFPSLRLLYIMD  1000
PG05 IIMLHDQILFILTIIVGVVGSFIEVIWTLIPAFILILIAFPSLRLLYIMD  1000
PG06 IIMLHDQILFILTIIIGVVGSFIEVIWTLIPAFILILIAFPSLKLLYIMD  1000
PG07 IIFFHDQIMFILTIILTTVGTLIEIIWTLIPAGILVFIAFPSLKLLYLMD  1000
PG08 IIFFHDQIMFILTIIVTIVGTLIEIIWTLVPACVLIFIAFPSLKLLYLMD  1000
PG09 VIFFHDQIMFILIIIISIVGTLIEIIWTLIPAGVLVFIAFPSLKLLYLMD  1000
PG10 IVFFHDQIMFLLVIIVIVVGTLLEIVWTLIPAVILVFIASPSLKLLYLMD  1000
PG11 IVFLHDQIMFVVPVISVLVGTVIEVVWTVIPAIILIFIAFPSLKLLYLMD  1000
PG12 ILFLHNQIMFILITIITVVGATIEIVWTIIPAIILVFIAFPSLKLLYLMD  1000
PG13 IIFFHDQVMFILIIIITTVGTLLEIIWTIVPAIILILIAFPSLKLLYLMD  1000
PG14 IIFFHDQIMFLLIIIVIVVGTLLEIVWTLIPAVILIFIASPSLKLLYLMD  1000
PG15 LIFFHDHALLVVILITSLVGQAIETVWTVIPAIILVAIALPSIRLLYLID  1000
PG16 LIFFHDHAMLILIMIISLLSQEIEIVWTILPAVVLIFLAFPSLQLLYLLD  1000
PG17 LIYFHDHTLIIILTLIITVVSQQIETAWTLIPAIILIFIALPSLRLLYLMD  1000
PG18 LTYFHDYALIVLTLITILVGQELETIWTVIPALILILIALPSLQLLYLMD  1000
```

```
PG01 EIIEPQVTVKAIGNQWYWSYEYTDYPLRQLNVDNYLVLPVDTSIRLLITA  1050
PG02 EVIDPAITIKVVGHQWYWSYEYSDYDLRLLEVDNRVVLPKNTHVRVIVTS  1050
PG03 ECGFSAITIKVTGHQWYWSYSYEDYDLRLLEVDNRLILPVDTNVRVLITA  1050
PG04 ECGFSAITIKVTGHQWYWSYSYEDYDLRLLEVDNRLILPVDTNVRVLITA  1050
PG05 EGVFSAITIKVTGHQWYWSYSYEDYDLRLLEVDNRLIIPVDTNVRVLITA  1050
PG06 EGVFSAITIKVTGHQWYWSYSYEDYDLRLLEVDNRLIIPVDTNVRVLITA  1050
PG07 EVIDPALTIKAIGHQWYWSYEYSDYDLRLLEVDNRLIVPIQTQVRVLVTA  1050
PG08 EVIDPALTIKAIGHQWYWSYEYSDYDLRLLEVDNRLIVPIQTQVRVLVTA  1050
PG09 EVIDPALTIKVVGHQWYWSYEYSDYDLRLLEVDNRLVVPIQTQVRVLVTG  1050
PG10 EVVSPALTIKAIGHQWYWSYEYSDYDFRLLEVDNRLVVPINTHVRILVTG  1050
PG11 ETMEPAITIKAVGHQWYWSYEYSDYDNRLLEVDNCLVLPIQAHIRVLVTG  1050
PG12 EIVEPGVTVKAIGHQWYWSYEYSDYDLRLLEVDNRMIVPIDTHVRVLVTG  1050
PG13 EVMDPALTIKAIGHQWYWSYEYSDYDFRLLEVDNRLVVPINTHVRVLVTG  1050
PG14 EVVGPALTIKAIGHQWYWSYEYSDYDFRLLEVDHRLLVPINTHVRILVTG  1050
PG15 EIHNPALTIKVTGHQWYWSYEYSDLMYRLLDVDNRSQCPMIKAIRLMITS  1050
PG16 ELEEPALTIKVVGHQWYWSYEYSDFDYRLLEVDHRSVVPMKTKVRVLVTA  1050
PG17 EINDPHLTIKATGHQWYWSYEYTDYEPRLLETDARIIAPTHSPIRILVTS  1050
PG18 EVNDPFLTIKAFGHQWYWSYEYTDFNPRLLEVDNRLVLPMQNPIRVLVSS  1050
```

```
PG01 SDVIHSFAVPSLGVKMDAIPGRLNSVGMIISRPGVFYGQCSELCGYLHGF   1100
PG02 ADVIHSWAVPSLGVKIDAIPGRLNQTSFLAYRNGVFYGQCSEICGIGHSV   1100
PG03 ADVLHSWTVPSLAVKMDAVPGRINETGFLIKRPGIFYGQCSEICGANHSF   1100
PG04 ADVLHSWTVPSLAVKMDAVPGRINETGFLIKRPGIFYGQCSEICGANHSF   1100
PG05 ADVLHSWTVPSLAVKMDAVPGRINETGFLIKRPGIFYGQCSEICGANHSF   1100
PG06 ADVLHSWTVPSLAVKMDAVPGRINETGFLIKRPGIFYGQCSEICGANHSF   1100
PG07 ADVLHSFAVPSLSVKIDAVPGRLNQTSFFIKRPGVFYGQCSEICGANHSF   1100
PG08 ADVLHAFAVPSLGVKVDAVPGRLNQTSFFLKRPGVFYGQCSELCGANHSF   1100
PG09 ADVLHSFALPSLSVKIDAVPGRLNQTSFFIKRPGVFYGQCSEICGANHSF   1100
PG10 ADVLHSFAVPALGIKTDAVPGRLNQAGIFIKRPGTFYGQCSEICGANHSF   1100
PG11 ADVIHSFAIPSLGIKMDAVPGRLNQSNMFMKRSGLFYGQCSEICGTNHSF   1100
PG12 ADVLHSFAVPSLGIKMDAVPGRLNQTGFLVKRPGIFYGQCSELCGANHSF   1100
PG13 ADVLHSFAVPALAVKMDAIPGRLNQTGFFIKRPGIFYGQCSEICGANHSF   1100
PG14 ADVLHSFAVPALGLKIDAVPGRLNQAGLFVKRPGLFYGQCSELCGANHSF   1100
PG15 DAVLHSWAVPSLGIKMDADPGRLNQSSLLVNMPGVFYGQCSEICGSGHSF   1100
PG16 ADVLHSWTVPSLGVKADAVPGRLNQLSFFANYPGVFYGQCSEICGANHSF   1100
PG17 DDVLHSWTIPALGLKMDAIPGRLNQITFYAPRSGVFYGQCSEICGANHSF   1100
PG18 ADVLHSWAVPSLGTKMDAVPGRLNQTTFFAARTGVFYGQCSEICGANHSF   1100

PG01 MPIGIKAVSTENYLNWHPYHLVDPSPWPILGGFAAFVFASGLIFSFKHIL   1150
PG02 MPIAIEVVDLENYCTYHPYHLVEPSPFPYMASFSALGLTVGAVMYFHSLL   1150
PG03 MPIVVESVSLSKYIDWHPYHLVDPSPWPFVGASGAFLFTSGAVILFHYLA   1150
PG04 MPIVVESVSLSSYIDWHPYHLVDPSPWPFVGASGAFLFTSGAVILFHYLA   1150
PG05 MPIVVEAVSLSKYIDWHPYHLVDPSPWPFVGASGAFLFTSGAVILFHYLA   1150
PG06 MPIVVEAVSLSKYIDWHPYHLVDPSPWPFVGASGAFLFTSGAVILFHYLA   1150
PG07 MPIVIEAVSLDKYINWHPYHLVEPSPWPYIGACGAFFTTVGSVMYFHYVL   1150
PG08 MPIVIEGVSLEKYVSWHPYHLVKPSPWPYIGGCGALLTTVGAIVYFHYVL   1150
PG09 MPIVIEAVSLEKFVNWHPYHLVDPSPWPYIGACGAFFTTVGGVMYFHYVL   1150
PG10 MPIVIEAVSLDRYINWHPYHLVEPSPWPCLGAGGAFFITVGSVMYFHYIM   1150
PG11 MPIAVRGVSLDTFINWHPYHLVDPSPWPYVSACGAFFLTLGSVIYFHYIM   1150
PG12 MPIVIEAVSMDKYISWHPYHLVDPSPWPYTGSVGALLITVGSVLYFHYLM   1150
PG13 MPIVIEAVSLDKYINWHPYHLVDPSPWPYVGACGALFITVGSVVYFHYVL   1150
PG14 MPIVIEAVSLDRYINWHPYHLVEPSPWPCLGAGGAFFITMGSVVYFHYVM   1150
PG15 MPIVIEAVGESDFLKWHPYHLVNISPWPLATGMGAFAMTSGLVKWFHSLF   1150
PG16 MPIVLEVVDSSSFIKWNPFHLVEFSPWPLVGSMGAFCLTVGLAAWFHGLV   1150
PG17 MPIVLETVPLEEFEAWHPFHLVDQSPWPLTGATAALFTTVGIMVWFHFLT   1150
PG18 MPIVIESVPFNTFENWHPYHLVDQSPWPLTGAFSGLMMTSGNVLWFHTLT   1150

PG01 VLGLISVLLVTFGWFRDITREGGKHTTKVQSGLMLGVLLFLGSEVLLFIS   1200
PG02 SISAILLTYVAYLWWRDIVREQGHHTIVVQKGLRLGMLLFIVSEVMFFFG   1200
PG03 LTGLFLIIITASAWWRDVIREQGHHTQVVVRGLKLGMLLFILSEVCLFLA   1200
PG04 LTGLFLIIITASVWWRDVIREQGHHTQIVVRGLKLGMLLFILSEVCLFFA   1200
PG05 LIGLLLIATAASAWWRDVIREQGHHTQIVVRGLKLGMLLFILSEVCLFFA   1200
PG06 LTGLFLIIITASAWWRDVIREQGHHTQIVVRGLKLGMLLFILSEVCLFFA   1200
PG07 ILGLITILFTMVVWWRDVIREQGHHTLIVKQGLKYGMILFILSEVCLFFS   1200
PG08 SFGLVIIVVTMVVWWRDVIREQGHHTLIVKQGLKYGMILFILSEVCLFFS   1200
PG09 IIGLTVIVFTMIVWWRDVIREQGHHTLIVKQGLKYGMLLFILSEVCLFFS   1200
PG10 YLGVLVVVIIMFVWWQDVIREQGFHSLVVKQGIKYGMLLFILSEVLFFFS   1200
PG11 LLGLLILGTTFVVWCRDIIREQGHHTEIVKKGLKIGMLLFIVSEICFFVS   1200
PG12 YLGAITLILTMIVWWRDVIREQGHHTQIVKRGLRYGMILFITSEVCFFFA   1200
PG13 LMGAITLSLTMIVWWRDVIREQGLHTMVVKQGLKYGMLLFILSEVLFFFS   1200
PG14 YVGVLVVMVIMFVWWQDVIREQGYHSLVVKQGIRYGMLLFILSEVLFFFS   1200
PG15 FTGINNNHICFDQWWRDVSREQGMHSTKVGFGLRWGMILFIISEVFFFVS   1200
PG16 WVGVILILLTMVQWWRDVIREQGYHTLRVSRGLRWGMILFITSEVLFFFA   1200
PG17 LFGVVMVILTMFQWWRDVVREEGQHTSEVGSGLRWGMILFITSEVLFFFA   1200
PG18 LVGFLLLITNMVNWWRDIIRKQGSHTAIVNKGMRYGMILFITSEVCFFFA   1200
```

```
PG01 FFWAYFHSSLSPDIVLGGVWPPSVDPWSIPLLGSIILLSSGATCTVAHNA   1250
PG02 FFWAFFHSSLGPEISIGAVWPPPIEAMGVPLLNTAILLTSGATVTWSHHS   1250
PG03 FFWAFFHSSLAPSVELGSVWPPALDPFAVPLLNTTILLSSGATVTWAHHA   1250
PG04 FFWAFFHSSLAPSVELGSVWPPALDPFAVPLLNTTILLSSGATVTWAHHA   1250
PG05 FFWAFFHSSLAPSVELGGVWPPALDPFAVPLLNTTILLSSGATVTWAHHA   1250
PG06 FFWAFFHSSLAPSVELGGLWPPALDPFAVPLLNTTILLSSGATVTWAHHA   1250
PG07 FFWAFFHSSLAPTIEIGAVWPPPLNPFSVPLLNTAVLLSSGATVTWAHHG   1250
PG08 FFWAFFHSSLVPTVEIGAVWPPPLNPFSVPLLNTVVLLSSGATVTWTHHA   1250
PG09 FFWAFFHSSLVPTIEIGAVWPPPLDPFSIPLLNTAVLLSSGATVTWAHHG   1250
PG10 FFWAFFHSSLAPAVELGVVWPPPLNSFSVPLLNTAVLLSSGATVTWAHHA   1250
PG11 FFWAFFHSSLAPAIELGALWPPILNPFSVPLLNTAILLSSGATVTWAHHA   1250
PG12 FFWAFFHSSLSPTIELGAVWPPVLDPFSVPLLNTAILLSSGATVTWAHHA   1250
PG13 FFWAFFHSSIAPNIELGAVWPPPLNPFSVPLLNTAVLLSSGATVTWAHHA   1250
PG14 FFWAFFHSSLAPAVELGVVWPPPLNPFSVPLLNTAVLLSSGATVTWAHHA   1250
PG15 FFWGFFHSSLSPNIEVGALWPPEVESFLSALLNTSILLASGVTVTWAHHA   1250
PG16 FFWAYFHSSLAPCLEIGSCWPPPLNPFQVPLLNTAVLLASGVSVTWAHHS   1250
PG17 FFWAFFHSSLAPTIELGTCWPPPINPFQVPLLNTAVLLASGVTVTWAHHS   1250
PG18 FFWAFFHSSLAPSVEIGVAWPPPLNPFLVPLLNTGVLLSSGVTLSWSHHS   1250

PG01 LINGAVATMFATVVLGFLFVGLQATEYYVSEFSIADSIFGTVFYMTTGLH   1300
PG02 IIAGTILALILTVVLAFIFTILQGMEYDEATFTIADSVYGSVFYMLTGFH   1300
PG03 IIGGAVIGLLMTVLLGIIFTGLQAFEYKEASFAISDSVYGSTFFMLTGTH   1300
PG04 IIGTAVIGLLMTILLGIIFTGLQAFEYKETSFAISDSVYGSTFFMLTGTH   1300
PG05 IIGGAVIGLLMTILLGIIFTGLQAFEYKEASFAISDSVYGSTFFMLTGTH   1300
PG06 IIGGAVIGLLMTILLGIIFTGLQAFEYKEASFAISDSVYGSTFFMLTGTH   1300
PG07 IISGAIISLALTVVLGFIFTALQAMEYYEAPFTISDSVYGSTFFVTTGAH   1300
PG08 IISGAIIGLVFTVFLGILFTGLQAMEYYEAPFTISDSVYGSTFFLTTGAH   1300
PG09 IISGAIRGLALTVILGLIFTGLQAMEYYEAPFTISDSVYGSTFFVTTGAH   1300
PG10 IISGAILGLSLTVFLGVLFTGLQGIEYYEAPFTISDSVYGSTFFMATGFH   1300
PG11 IINGAIVGLVSTVLLGMIFTALQIVEYIEAPFAISDSVYGSTFFMATGFH   1300
PG12 IVSGAIQSLTCTVILGIQFTALQAMEYYEAPFTISDSVYGSTFFMATGFH   1300
PG13 LISGAINGLTITVVLGLIFTGLQAMEYYEAPFAISDSVYGSTFFVATGFH   1300
PG14 IISGAIRGLSFTIFWGILFTGLQAIEYYEAPFAISDSVYGSTFFVATGFH   1300
PG15 LMENCLQGLLFTVLLGLYFSFLQGLEYMEASFTIADSIYGSTFFLATGFH   1300
PG16 LIDGANISLLTTVILGAYFTFLQAGEYLETSFTIADSCYGSTFFVATGFH   1300
PG17 IIAAAIQALLITVLLGLYFTALQIFEYYQAPFTIADAVYGSTFFVATGFH   1300
PG18 ILAGSIQALFLTVALGSYFTALQAWEYIDAPFTIADSVYGSTFFVATGFH   1300

PG01 GTHVIIGAIFLTVMLVRMVRDQFTHHLGLEFSLWYWHMVDVIWLFVFLTY   1350
PG02 GFHVLVGTIFLTVCLFRIIRSHYTHHNGFEAAIWYWHFVDFVWLGLFVIV   1350
PG03 GLHVLVGTTFLIVCFIRLLSNQFTHHLGLEAAIWYWHFVDVVWLFLYVFV   1350
PG04 GLHVLVGTTFLLVCFIRLLSNQFTHHLGLEAAIWYWHFVDVVWLFLYVFV   1350
PG05 GLHVLVGTTFLIVCFFRLLANQFTHHLGLEAAIWYWHFVDVVWLFLYVFV   1350
PG06 GLHVLVGTTFLIVCFFRLLANQFTHHLGLEAAIWYWHFVDVVWLFLYVFV   1350
PG07 GGHVLIGSTFLLVCLYRLINHQFTHHFGFEAAAWYWHFVDVVWLFLFTFM   1350
PG08 GAHVLIGSSFLLVCLIRLIYYQFTHHFGFEAAVWYWHFVDVVWLFLFIFM   1350
PG09 GGHVLIGSSFLLVCLYRLVTHHFTHHVGFEAAAWYWHFVDVVWLFLFTFM   1350
PG10 GFHVLIGTTFLIICLVRLKLNQFTQHVGFEAASWYWHFVDVVWLFLYLCV   1350
PG11 GFHVLVGTTFLLVCLLRLVFHQYTHHFGFEAASWYWHFVDVVWLFLYVCI   1350
PG12 GFHVIIGTIFLTVCLTRLIGYHYTHHFGFEAASWYWHFVDVVWLFLYVCI   1350
PG13 GMHVIIGTTFLAVCLARLVYHQFTHHLGFEAASWYWHFVDVVWLFLYICI   1350
PG14 GLHVIIGTTFLTVCLVRLKSNQLTKHVGFEAASWYWHFVDVVWLFLYLCI   1350
PG15 GLHVLIGTIFLMICILRHAKCYFSHHFGFEAAAWYWHFVDVVWLFLYLSI   1350
PG16 GFHVLVGSLFLLVTLWRNFSCHFSHHFGFEAAAWYWHFVDVVWLFLYISI   1350
PG17 GLHVIIGTTFLLICLVRQLKFHFSHHFGFEAAAWYWHFVDVVWLFLFISI   1350
PG18 GLQVIIGTTFLMVCLFRTAGRHFSHHFGFEAAAWYWHFVDVVWFVLYWLI   1350
```

```
PG01  YWWGSVLAVAFMTLAERKLMGAIQRRIGPNSVGFLGLLQPFFDGVKLILK  1400
PG02  YWWGSLIAVAYLTLAERKVLASMQLRKGPTAVGVFGLLQPLADGLKLFVK  1400
PG03  YWWGSLLSVAYLTLAERKVLGLMQNRKGPAIVGPWGLVQPIADGVKLLTK  1400
PG04  YWWGSLLSVAYLTLAERKVLGLMQNRKGPAIVGPWGLVQPIADGVKLLTK  1400
PG05  YWWGSLLSVAYLTLAERKVLGLMQNRKGPAIVGPWGLVQPIADGVKLLTK  1400
PG06  YWWGSLLSVAYLTLAERKVLGLMQNRKGPAIVGPWGLVQPIADGVKLLTK  1400
PG07  YWWGSLVSIAYLTLAERKVLGYIQCRKGPNVVGVYGLLQPLADGLKLFTK  1400
PG08  YWWGSLISIAYLTLAERKVLGYMQARKGPNVVGVYGLLQPLADGLKLFIK  1400
PG09  YWWGSLISIAYLTLAERKVLGYIQCRKGPNVVGLYGLLQPIADGLKLFTK  1400
PG10  YWWGSLITVAYLTLAERKVLGYMQARKGPNVVGVSGLAQPFADGLKLFTK  1400
PG11  YWWGSLIAVAYLTLAERKVLGYMQSRKGPNVVGIYGLLQPLADGVKLFSK  1400
PG12  YWWGSLVAIAYLTLAERKVLGYMQARKGPNVVGVYGLLQPLADGLKLFTK  1400
PG13  YWWGSLVAVAYLTLAERKVLGYMQARKGPNVVGVYGLLQPLADGIKLFTK  1400
PG14  YWWGSLIAVAYLTLAERKVLGYMQARKGPNVVGVAGLVQPFADGVKLFTK  1400
PG15  YWWGELVSVAFLTLLERKILGYIQLRKGPNKVGFLGILQPFSDGVKLFCK  1400
PG16  YWWGSLLAVAFFTLLERKGLGYIQFRKGPNKVSIFGLAQPLADALKLFLK  1400
PG17  YWWGSLLSVAFITLGERKALGYMQARKGPTLVGPYGLLQPIADGVKLFIK  1400
PG18  YWWGALLSVAFLTLVERKVLGYMQFRNGPNVVGPFGLLQPFADGMKVFIK  1400

PG01  ETVLPLESNHWIFVFAPFFTFYLALLNWLVIPLDKGLMDLEASILYLLAV  1450
PG02  EAVLPTHANMIIFVVSPVAAFTLALISWAVIPYNEGIADVNIGLLYIFAV  1450
PG03  ELIIPSQANKFMYYIAPIISFTLAILAWAIVTMDQGLSDLDIGVLYFFAI  1450
PG04  ELIIPSQANKFMYYIAPIISFTLAILAWAIVTMDQGLSDLDIGVLYFFAI  1450
PG05  ELIIPSQANKFLYYIAPIISFTFAILAWAIVTMDQGLSDLDVGVLYFLAI  1450
PG06  ELVIPSQANKFLYYIAPIISFTLAILAWAIVTMDQGLSDLDVGVLYFLAI  1450
PG07  EIIIPNHANNLIYILAPILSLTLALVAWGVIPYGQGLSDLGIGILYLFAV  1450
PG08  EIIIPNHANMFIYIVAPILSLTLAFIAWGVIPYGPGLSDLGIGVLYLFVV  1450
PG09  EIIIPNHANFFIYILAPILSLTLAFIAWGVIPYNQGLSDLEVGILYLFAV  1450
PG10  EMVVPHQTNLFIYIVAPVLSFTLALIVWGVVPYERGISDLKIGVLYILAV  1450
PG11  EMTVPNHSSLILYFISPILFLTLALVLWGVLPYTLNYSELSLGVLFILAI  1450
PG12  EMVIPNHANLSVYIVAPILSLTLAFLAWGVIPFSPGLADINVGILYIFAI  1450
PG13  ELVIPHYANLFIYVAAPVLSFTLALIAWGVIPYDKGISDLKIGILFTLAV  1450
PG14  EMIIPHHTNLFIYIVAPMFSFTLALVVWGVVPYERGISDLKIGILYVLAI  1450
PG15  EVSLPLVSNFMPYLVAPVFSLFLSFFLWTLVPFISYGAKFNLSFLLVICA  1450
PG16  EENNLYNSNKLPYYTAPVLSLILSLCLWSLYWSDFTLWYFNFGVLFFLCT  1450
PG17  EPIKPSSAASWLFLATPILALTIALILWVPMTFPHQFSSINLSLIFILTL  1450
PG18  EELKPVNSSPYLFFFSPLLFLALALLLWNFMPVHTPTLDLQLSLLLVLGL  1450

PG01  SSLGVYGIIYSGWAANSKWTLLGSLRSTAQMVSYEVSMSLIVLTVVYAGA  1500
PG02  SSISVYAILMSGWASNSKYAFFGAIRAAAQMISYEVSMGLILLSVILCVG  1500
PG03  SSLSVYAILLSGWASNSRYPFLGAIRCAAQMISYEVAIGLIIISVILCVG  1500
PG04  SSLSVYAILLSGWASNSRYPFLGAIRCAAQMISYEVAIGLIIISVILCVG  1500
PG05  SSISVYAILLSGWASNSRYPFLGAIRCAAQMISYEVAIGLIIISVILCVG  1500
PG06  SSLSVYAILLSGWASNSRYPFLGAIRCAAQMISYEVAIGLIIISVILCVG  1500
PG07  SSISVYAVLMSGWSSNSKYAFLGAIRAAAQMISYEVSIGLIIISVVLCVG  1500
PG08  SSISVYAILMSGWSSNSKYAFLGAIRAAAQMISYEVSIGLIIIAVILCVG  1500
PG09  SSISVYAILMSGWSSNSKYAFLGAIRAAAQMISYEVSIGLIIISVVLCVG  1500
PG10  SSISVYAILMSGWASNSKYAFLGAIRAAAQMISYEVSIGLIIISVLLCVG  1500
PG11  SSVSVYAILMSGWASTSKYAYFGSLRAAAQMISYEVSMGLILISVVICIG  1500
PG12  SSMGVYAILMSGWGSNSKYAFLGAIRAAAQMISYEVCIGLILISVILCAG  1500
PG13  SSISVYAILMSGWASQSKYAFLGAIRAAAQMISYEVSIGLIIITVILCVG  1500
PG14  SSISVYAILMSGWASNSKYAFLGAIRAAAQMISYEVSIGLIIISVLLCVG  1500
PG15  MSVSVYSIMVAGWSSNSKYSLLGSIRAGAQTISYEVSLIIIILSPLMLFK  1500
PG16  SALSVYGTMIAGWASNSKYALLGALRAVAQTISYEVTLFLVLLSSAALSS  1500
PG17  SSLAVYTILGSGWASNSKYALLGAIRAVAQTISYEVSLGLTILCIIVLAG  1500
PG18  SSLSVYAILGSGWASNSKYSLLGAIRAVAQTISYEISLALILLSLIIFSS  1500
```

```
PG01  SVNMFIWPLWPIAIIAFIAGLAETNRAPMDLPEAESELVAGFMTEHSAIS  1550
PG02  DINWFIIPLFPAFIMFLVSALAETNRAPFDLTEGESELVSGYNVEYSAIF  1550
PG03  SLNWLIIPLFPAAIMFMVASLAETARAPFDLTEGESELVSGFNVEYSSLS  1550
PG04  SLNWLIIPLFPAAIMFMVASLAETARAPFDLTEGESELVSGFNVEYSSLS  1550
PG05  SLNWLIIPLFPAAIMFMVASLAETARAPFDLTEGESELVSGFNVEYSSLS  1550
PG06  SLNWLIIPLFPAAIMFMVASLAETARAPFDLTEGESELVSGFNVEYSSLS  1550
PG07  SLNWFLIPLFPITIMFFVSALAETNRVPFDLTEGESELVSGFNVEYSSMS  1550
PG08  SLSPLILPLFPAAIMFFVSVLAETNRVPFDLTEGESELVSGFNVEYSSMS  1550
PG09  TLNWFIVPLFPAGIMFFVSALAETNRVPFDLTEGESELVSGFNVEYSSMS  1550
PG10  SLSWFFFPLFPVTIMFFVSALAETNRAPFDLTEGESELVSGYNVEYASMS  1550
PG11  SLNLFIFTLIPAGIMFMVSLIAETNRAPFDLTEGESELVSGYNVEYSSMS  1550
PG12  SLNWYIIPLFPAALMFFASALAETNRAPFDLTEGESELVSGYNVEYSSMS  1550
PG13  SLNWFFFPLFPVAMMFFASALAETNRAPFDLTEGESELVSGYNVEYASMS  1550
PG14  SLSWFFLPLFPVTIMFFASALAETNRAPFDLTEGESELVSGYNVEYASMS  1550
PG15  KLDWPLYLCLPLGLCWFTTILAETNRTPFDLAEGESELVSGFNTEYMGVG  1550
PG16  SLNWNSILMTPLLLMWIVVCLAETNRAPFDFAEGESELVSGFNIEYGSGS  1550
PG17  NFNWFILTCAPVSIMWYISTLAETNRSPFDLAEGESELVSGFNVEYAGGP  1550
PG18  SFNWFSLSCLPLFYIWFVSTLAETNRAPFDLTEGESEIVSGYNVEYAGGP  1550

PG01  FTFLFLAEYTNIITMSTVISLYFFIWVRASLPRLRFDQLLRLGWVYLLPI  1600
PG02  FTLFFLAEYTHIIFMSILTSLLFFVWVRATFPRFRYNTLMELMWKSYLPL  1600
PG03  FALFFLAEYSNIILMSCMMTIFFFLWSRASFPRIRYDQLMKLLWKTYLPL  1600
PG04  FALFFLAEYSNIILMSCMMTIFFFLWSRASFPRIRYDQLMKLLWKTYLPL  1600
PG05  FALFFLAEYSNIILMSCMMTIFFFIWSRASFPRIRYDQLMKLLWKTYLPL  1600
PG06  FALFFLAEYSNIILMSCMMTIFFFIWSRASFPRIRYDQLMKLLWKTYLPL  1600
PG07  FALFFLAEYCHIILMSTFGVILFFIWIRASYPRIRYDQLMALLWKSYLPL  1600
PG08  FALFFLAEYCHIILMSTFAVLLFFIWIRATYPRIRYDQLMTLLWKSYLPL  1600
PG09  FALFFLAEYCHIILMSAFGVILFFIWIRASYPRIRYDQLMALLWKSYLPL  1600
PG10  FALFFLAEYAHIILMSCLTIIFFFIWVRASFPRIRYDQLMSLLWKAYLPL  1600
PG11  FALLFLAEYANIIFMSALFSLIFFVWVRASYPRIRYDQLMMLLWKSYLPL  1600
PG12  FALFFLAEYGHIILMSCLISLLFFVWVRASFPRMRYDQLMYLLWKSYLPF  1600
PG13  FALFFLAEYAHIILMSCLTTILFFIWVRASFPRMRYDQLMALLWKSYLPL  1600
PG14  FALFFLAEYAHIILMSCLTTILFFIWVRASFPRIRYDQLMALLWKAYLPL  1600
PG15  FALIMLSEYASILFMSLLFSVVFYLWSRGSYPRYRYDNLMHLCWKSLLPT  1600
PG16  FALLFLAEYASILFMSMMTTILFFIWVRGTFPRLRYDLLMKLTWLSFLPV  1600
PG17  FALFFLAEYSNIIFMNALSTILLFLWVRASYPRYRYDQLMHLAWKTFLPL  1600
PG18  FVLFFIAEYANIILMNYFSVVLFVLWVRAAYPRFRYDQLMFLTWKSYLPL  1600

PG01  LIGYDWLISLIGFEFLNLSTYLILKYLLMSAFFTTLLLLSISLLLLTLSF  1650
PG02  SLSKDLIIMYLSIETLSLSLYILLKYLIMGGLSSGILLFGCALICIAIMF  1650
PG03  SLSANWLTIYLALELQTLALFILLKYWILGAMSSGFYLFGCALITVAILF  1650
PG04  SLSANWLTIYLALEFQTLALFILLKYWILGAMSSGFYLFGCALITIAILF  1650
PG05  SLSANWLTIYLALEFQTLALFILLKYWILGAMSSGFYLFGCALITVAILF  1650
PG06  SLSANWLTIYLALEFQTLALFILLKYWILGAMSSGFYLFGCALITVAILF  1650
PG07  SLSVNWLSIYLAIELQTLTLFILLKYFVLGAVSSGVFLFGCALITISLLF  1650
PG08  SGSINWFSIYLAIELQTLTLFILLKFFVLGAVSSGLFLFGCVLVTISLLF  1650
PG09  SLSINWLSIYLAIELQTLTLFILLKYFVLGAVSSGLFLFGCTLITISLLF  1650
PG10  SLSSNWLSVYLAIELSTLSLFILLKYFVLGALSSGLFLFGCALIVVSLLF  1650
PG11  SLTTNMLALYLCIELQSLSIFILLKYFVLSSISSGLLLLGSSLILVALLF  1650
PG12  SLSTNLISIYLALEMQTLCMFILLKYFVLGALSSGLFLFGCALITVSLLF  1650
PG13  SLASNWLSVYLAIELPTLSLFILLKYFVLGALASGLFLFGCALITGALLF  1650
PG14  SLSSNWLSVYLAIELSTLSFFILLKYFVLGALSSGLFLFGCALIVVALLF  1650
PG15  SLSHSWLGLWLSMEMNSLSFIPILKYFLIQSVASVIFLASILNIPFALLI  1650
PG16  SLSIHWFGVWLGLELNLMGFIPVVKYFLVQAVGSALFLFGLMLIFFGLLM  1650
PG17  SLSSHWFFIWLGLEINTLAFLPLTKYFLIQALASALLLLGGLLLLIAIAL  1650
PG18  SISENWFIIWVGLELSTLALVPINKYFLVQASSAALLLNGALGLSIALAF  1650
```

```
PG01 KLGLVPVHLWVPDVYDGLPMELVLWIGTLPKLAILFFLSFLLAAISLGAQ 1700
PG02 KLAAAPFHMWAPDVYEGSPTIITAFFAIVPKIAILTLLSVIIGSIGAVNQ 1700
PG03 KLAAAPFHMWTPDVYEGAPTPTTALIAIIPKFTVYILLSLVVGAFGALNQ 1700
PG04 KLAAAPFHMWTPDVYEGAPTPTTALIATIPKFTVYILLSLVVGAFGALNQ 1700
PG05 KLAAAPFHMWTPDVYEGAPTPTTALIAIIPKYVAFVLLSLIVGAFGALNQ 1700
PG06 KLAAAPFHMWTPDVYEGAPTPTTALIAIIPKYVAFVLLSLIVGAFGALNQ 1700
PG07 KLSAAPFHMWAPDVYDGAPTTTTALLAIVPKVGVFSILSIIYGAIGALNQ 1700
PG08 KLSAVPFHMWAPDVYEGSPTIITALLTTVPKIGVFSILSIVYGAIGALNQ 1700
PG09 KLSAAPFHMWAPDVYEGAPTIITALLATVPKVAVFSILSIVYGSIGALNQ 1700
PG10 KLSAAPFHMWAPDVYDGAPTTTTALLATVPKVGYFSILSMVVGAIGALNQ 1700
PG11 KLAASPFHFWLPDVYQGSNNNTLLLLGTLPKISVLGIFSLIIGCIGAINQ 1700
PG12 KVSAAPFHMWAPDVYEGAPTWVAALLSIVPKLGVLAIISLVIGAIGALNQ 1700
PG13 KLSAAPFHMWAPDVYDGAPTTTTALLATVPKVGVFSILSMVVGALGALNQ 1700
PG14 KLSVAPFHMWAPDVYDGASTTTTALLAIVPKVGVFSILSLLVGALGALNQ 1700
PG15 KIGAAPFHMWLVSISKSMSWKVLSLLMTFQKIGPLLGLSAFIGGLGGINQ 1700
PG16 KLGAAPFHFWVPSVVAGLSWMSNFLLLTVQKIAPLFMVSSLFGGVGGVNQ 1700
PG17 KMAIPPCHNWLPDVLQGLPLLTGLILSTWQKLAPFYILAALVGGWGGLNQ 1700
PG18 KIGLAPVHFWFPDVLQGLPFFQGLIIATWQKIAPLILMSVLIGGWGGLNQ 1700

PG01 YRLKRFLAFSAIGHMGIMITGLTMAAVPPFSGFYAKLLVLTLEKVSVYEC 1750
PG02 TKFKRLLAYSAISHMGFILLGFSIAGIPPLAGFISKYYILDYEKVQQYEC 1750
PG03 TRIKRLLAYSGIGHMGFILIGLSLAGIPPLLGFLGKWFILDSEKSSIYEC 1750
PG04 TRIKRLLAYSGIGHIGFILIGLSLAGIPPLLGFLGKWFILDSEKSSIYEC 1750
PG05 TRVKRLLAYSGIGHIGFILIGLSLAGIPPLLGFLGKWFILDSEKSSIYEC 1750
PG06 TRVKRLLAYSGIGHIGFVLLGLSLAGIPPLLGFLGKWFILDSEKSSIYEC 1750
PG07 TKIKRLLAYSGIGHIGFILFGLSTAGVPPLAGFLSKWLILDSEKVSVYEC 1750
PG08 TKVKRLLAYSGIGHMGFILLGLSTAGIPPLAGFLSKWLVLDSEKVSVYEC 1750
PG09 TKIKRLLAYSGIGHMGFILFGLSIAGIPPLAGFLSKWLILDSEKVSVYEC 1750
PG10 TKVKRLLAYSGVGHMGFVLWGLSIAGIPPLVGFWGKWLVLDREKVSAYEC 1750
PG11 SKTKRLLAYSSIVGMGFILLGLSVAGIPPLGGFFSKWLVLDKEKVSVYEC 1750
PG12 TRIKRLLAYSGIGHIGFVLLGLSTAGIPPLIGFLTKWYIIDMEKVSVYEC 1750
PG13 TKIKRLLAYSGIAHMGFVLWGLSIAGVPPLIGFFSKWWILDREKVSAYEC 1750
PG14 TKVKRLLAYSGVGHMGFVLLGLSIAGIPPLVGFLGKWLVLDREKVSAYEC 1750
PG15 SNLRLIMAFSSVSHLSWLMVNLSLAGLPPFLGFFIKWMSLDREKSSPFEC 1750
PG16 TSVRALIAYSSILHMGWMLIGLSLGGLPPLLGFFGKWLVLNREKSSPFEC 1750
PG17 TQIRKLLAFSSIAHIGWTIAILSLAGLPPLTGFLGKWLIINLEKLSPYEC 1750
PG18 TQVRKILAFSSIGNMGWLVITLSLGGLPPLTGFILKFTSLDSEKSSPYEC 1750

PG01 GFDPYSDARLKFDIIYYLVAILFLIFDLEIVFLFPFFVVLTIGYLYELNS 1800
PG02 GFNPFSDSRQPFEVRFFLVGILFIIFDLEISFLFPFLGILTIGLVYEWLK 1800
PG03 GFDPFGKIRTPFAIKFVLVGILFMIFDIEVSFLFPFLVILSLGLAYEWLK 1800
PG04 GFDPFGKIRTPFAIKFVLVGILFMIFDIEVSFLFPFLVILALGLAYEWLK 1800
PG05 GFDPFGKIRTPFTIKFVLVGILFMIFDIEISFLFPFLIVLTIGLAYEWIK 1800
PG06 GFDPFGKIRTPFTIKFVLVGILFMIFDIEISFLFPFLIVLAIGLAYEWIK 1800
PG07 GFDPFGSSRVPFSVKFFLVGILFMIFDLEISFLFPFLIILTIGLIYEWVK 1800
PG08 GFDPLGSSRTPFSVKFFLVGILFLVFDLEISFLFPFLVILTIGLIYEWVK 1800
PG09 GFDPFGSSRVPFSVKFFLVGILFLIFDLEISFLFPFLIILTIGLVYEWAK 1800
PG10 GFEPFGIPGRLFQFRFFLVGILFLIFDLEISFLFPFLGVLTLGLIYEWIK 1800
PG11 GFAPYDNPGNPISIRFFLIGILFLVFDLEISLLLPFVFILTWGLLYEWIK 1800
PG12 GFDPFDNPGNPFSVRFFLIGILFLIFDLEISFLFPFLFILTLGLVYEWIE 1800
PG13 GFDPFGTPGRPFSIRFFLIGILFLIFDLEISFLFPFLAVLTLGLVYEWLK 1800
PG14 GFEPFGVPGRPFSVRFFLVGILFLIFDLEISFLFPFLGILTLGLIYEWIK 1800
PG15 GFDPLNSSRTPFSIRFFVITLIFLIFDVEIYLLLPFTFILVAGVFYEWSE 1800
PG16 GFDPKSSARLPFSMRFFLITVVFLVFDVEIVLLLPILVILIIGVLHEWSE 1800
PG17 GFEPHTSARLPFSLRFFLIAILFLLFDLEIALLLPVLAILTLGLIYEWVQ 1800
PG18 GFDPLNSARLPFSFRFFLVAILFLLFDLEIALLFPFMVILTLGLVFEWIN 1800
```

PG01 GSLDLSLVLIVLTTFLMPICLIVELVLPLLAIEALLMAVFLVLDVMIFYL 1850
PG02 GGLEWSLVFIILTTLLIPICILSSYLISFLVIEILLIGVFTILDIFGFYI 1850
PG03 GGLEWSIYFIILTGFIVPCSILISFLICMLILEFLLYAVFSVLDIFMFYI 1850
PG04 GGLEWSIYFIILTGFIVPCSILISFLICMLILEFLLYAVFSVLDIFMFYI 1850
PG05 GGLEWSIYFLILTAFIVPCSILISFLICMLVLEFLLYAVFSVLDIFWFYI 1850
PG06 GGLEWSIYFLILTAFIVPCSILISFLICMLVLEFLLYAVFSVLDIFWFYI 1850
PG07 GGLEWSIFFLILTALLTPICILISFLLCLLSIEILLMGVFTILDLVGFYV 1850
PG08 GGLEWSLFFLILTALLTPICVLISFLLCLLVIEILLMGVFTVLDLVGFYI 1850
PG09 GGLEWSIFFIILTALLTPICILISFLLCLLFMEILLMGVFTVLDLVGFYV 1850
PG10 GGLEWSLFFFVLTALLIPICILISFLLCLLFLEALLVGVFLVLDLLLFYL 1850
PG11 GGLEWSLPFILLTAILTPICIIISFLVFLLAIHLLLIGVFTSLNILLFYI 1850
PG12 GGLEWSIFFIVLSTLLTPICILVSFIMCLLGIELLLIGVFSTLDLLIFYV 1850
PG13 GGLEWSLFFLILTALLTPICILISFLLCLLFLEVLLMGVFSALDLLLFYI 1850
PG14 GGLEWSLFFFILTALLIPICVLISFLLCLLFLEVLLIGVFLVLDLLLFYI 1850
PG15 GALSWTLLTLNFSYFLFNSYQIKAYSTILVSLFVLLWLTFTTQSFILFYV 1850
PG16 GSLEWSVPLMMLSCWITGLMIMSSFLLNVMILNFIIIMVFTQKSLFSLYI 1850
PG17 GGLEWSSPLIILSAWLLPLSLLASYLTLLSSLVLSLIITFSALDLILFYI 1850
PG18 GGLEWSAPLIILSCWLAPIALIASFIIMIIVITGALIITFSSLELILFYI 1850

PG01 CFESVLIPLFYLMGKYKGRARRLSAALSLFLYTLAGSLIMLICIGLLYLE 1900
PG02 LFEGVLIPMYLIIGIWGSREQKITAGYYFFFYTLIGSVLMLIGILYLYSI 1900
PG03 LFEGVLIPMYLILGIWGSREEKMQAAYYFFFYTLIGSVLMLLCLFSLYNI 1900
PG04 LFEGVLIPMYLILGIWGSREEKMQAAYYFFFYTLIGSVLMLLCLFSLYNI 1900
PG05 LFEGVLIPMYLILGIWGSREEKMQAAYYFFFYTLIGSVIMLLCLFSLYNI 1900
PG06 LFEGVLIPMYLILGIWGSREEKMQAAYYFFFYTLIGSVIMLLCLFSLYNI 1900
PG07 LFEGILIPMFLIIGIWGSREEKVQASYYFFFYTFIGSVFMLLAMFTLYSY 1900
PG08 LFEGVLIPMVLIIGIWGSREEKVQAAYYFFFYTFIGSVFMLLAIFFLYDQ 1900
PG09 LFEGILIPMFLIIGIWGSREEKIQASYYFFFYTFIGSVFMLLGIFTLYSY 1900
PG10 LFEGILIPMFLLIGVWGSREEKVRASYYFFFYTFAGSVFMLLGLFQIYSV 1900
PG11 MFEGILIPMFLMIGIWGSRKEKERAAFYFFFFTLAGSLFMLLGIFTIYKH 1900
PG12 LFESILIPMFLIIGVWGARAEKIKAAYYFFFYTLVGSILMLLSIAVIYRI 1900
PG13 LFEGILIPMFLLIGVWGSREEKVRASYYFFFYTFVGSVFMLLGIFQLYSS 1900
PG14 LFEGILIPMFLLIGVWGSREEKVRASYYFFFYTFAGSVFMLLSIFQLYRN 1900
PG15 FFECSLIPTIILILGWGYQPERLPASYYFLFYTLLSSLPLLFIIIAHTSI 1900
PG16 FFEASLIPTLILILMWGYQPERLQAGMYMMIYTILGALPFLINIFFIYSH 1900
PG17 AFEATLVPTLIVITRWGNQPERLQAGTYFLFYTLFGSFPLLISLLSLLSL 1900
PG18 VVETTLIPTLILITRWGAQMERCQAGLYFMFYTLFGSLPLLIAIIAIYIS 1900

PG01 ASTKIPMIPFHLWLPEAHVESPTGGSILLAGILLKLGGYGFYYQPFVAVL 1950
PG02 SGTKIPKFPFHIWLPQAHVEAPVAGSVILAGILIKLGGYGFYFAPLVFIL 1950
PG03 HGTKIPIFPFHIWLPKAHVEAPVAGSVLLAGILLKLGGYGLNLAPAVIAM 1950
PG04 HGTKIPIFPFHIWLPKAHVEAPVAGSVLLAGILLKLGGYGLNLAPAVIAM 1950
PG05 HGTKIPIFPFHIWLPKAHVEAPVAGSVLLAGILLKLGGYGLNLAPAIIAM 1950
PG06 HGTKIPIFPFHIWLPKAHVEAPVAGSVLLAGILLKLGGYGLNLAPAIIAM 1950
PG07 AGTKIPKIPFHIWLPQAHVEAPVAGSVLLAGVLLKLGGYGFFFAPLIQTL 1950
PG08 VGTKIPKIPFHIWLPQAHVEAPVAGSVILAGILLKLGGYGFYFAPFIRIL 1950
PG09 AGTKIPKMPFHIWLPQAHVEAPVAGSVILAGVLLKLGGYGFYFGPLIQTL 1950
PG10 TGTKIPQIPFHIWLPQAHVEAPVSGSVILAGILLKLGGYGFYFAPLIIML 1950
PG11 TGTKIPKIPFHIWLPQAHVEAPVAGSVLLAGVLLKLGGYGFYFSPVIILL 1950
PG12 TGTKIPMIPFHIWLPLAHVEAPLAGSVILAGILLKLGGYGFYLAPIILTL 1950
PG13 VGTKIPQVPFHIWLPQAHVEAPVSGSVILAGILLKLGGYGFYFAPFVIML 1950
PG14 TGTKIPQIPFHIWLPQAHVEAPVSGSVILAGILLKLGGYGFYLAPLIVML 1950
PG15 YSSKLPVYFAHIWLPKAHVEAPVTGSMVLAAILLKLGGYGLYSETTLMGV 1950
PG16 NAHKLPIYSFHLWLPKAHVEAPVAGSMILAALLLKLGGYGIVLNYFIITL 1950
PG17 TSSKLPIYGLHLWLPKAHVEAPIAGSMILAAILLKLGGYGLFASEPIITF 1950
PG18 SSSNLPVYGFHLWLPKAHVEAPVAGSMILAAILLKIGGYGLALSLALIVF 1950

```
PG01 ALIGIVYAGLTAIRQIDMKKIIAYSSVAHMNLAMLGIFANNLLGIEGSIH 2000
PG02 GVLAVIYASLTTLRQTDLKRIIAYSSVSHMGLVSLGIFTLTANGIMGSIF 2000
PG03 GIVGIVYGSFATCRQIDVKRLVAYSSVAHMGLVSITLVTHDIIGLTGSIF 2000
PG04 GIVGIVYGSFATCRQIDVKRLVAYSSVAHMGLVSIALVTHDIIGLTGSIF 2000
PG05 GIIGIVYGSFGTCRQIDVKRLVAYSSVAHMGLASITLVTHDIIGLTGSIF 2000
PG06 GIIGIVYGSFGTCRQIDVKRLVAYSSVAHMGLASITLVTHDIIGLTGSIF 2000
PG07 SLLAIIYGSLTTCRQVDLKRIIAYSSVAHMGLVTLSLFSHTIQGLVAAVY 2000
PG08 SLLAIIYGSLTTCRQVDLKRIVAYSSVAHMGLVTLSLFSHTVQGLVAAVF 2000
PG09 SLFAIIYGSLTTCRQIDLKRIIAYSSVAHMGLVVLGLFSHTIQGLVAAVF 2000
PG10 GVVAIIYGGLLTCRQVDFKRLIAYSSVAHMGLVPLGLFTHTIEGLVAAVF 2000
PG11 GSIAVIYASLSTCRQTDAKKLVAYSSVAHMGLVTIAIFSRTSEGLVAAII 2000
PG12 SLIAVIYGSLTTCRQVDAKRLIAYSSVAHMGIVTIGLFTHTMEGLIASIF 2000
PG13 SVIAIIYGSLTTCRQVDLKRLIAYSSVAHMGLVTLGLFTHTIEGLVAAVF 2000
PG14 GVVAIIYGSLITCRQVDLKRLIAYSSVAHMGLVPLGLFTHTIEGLVAAVF 2000
PG15 CLMGGIFSCLICLRQSDVKSLIAYSSVAHMSFVILGMLMSCTYTNMSSIL 2000
PG16 GLVGGVLSSLICLRQSDMKALIAYSSVGHMGLMLAGLLSSFEFGLKMALL 2000
PG17 CIWGGLITSIICIRQTDMKALIAYSSVGHMSLVAAGIFSETTWGLNGALI 2000
PG18 CTWGALITSVICVRQTDLKALIAYSSVGHMSIVAAAIFSETSWGMNGALM 2000

PG01 MMVAHGLVSGALFYCVGLLYDRYHTRLVRYYRGLAIYMPNVAFPGSANFI 2050
PG02 LQLAHGLVSSALFIIVTVLYDRHHTRLVKYYRGMTVTMPNIAVPLSCNFV 2050
PG03 IMLAHGIVSSLLFILVTILYDRHHTRLIKYYRGLSITMPNVAVPPSGNFI 2050
PG04 IMLAHGIVSSLLFILVTILYDRHHTRLIKYYRGLSITMPNVAVPTSGNFI 2050
PG05 IMLAHGIVSSLLFILVTILYDRHHTRLIKYYRGLSISMPNIAAPPSSGFV 2050
PG06 IMLAHGIVSSLLFILVTILYDRHHTRLIKYYRGLSISMPNVAAPPSGGFV 2050
PG07 LMLAHGLVSSALFIAVTFLYERHYTRLVKYYRGMVITMPNASIPLSCNFI 2050
PG08 LMLAHGLVSSALFIAVTFLYERHHTRLVRYYRGMVITMPNASIPLSCNFV 2050
PG09 LMLAHGLVSSALFIIVTLLYDRFHTRLVKYYRGIVVTMPNASIPLSCNFV 2050
PG10 MMLAHGFVSSALFIAITYLYERHHTRLIKYYRGVTLTMPNMGIPLSCNFV 2050
PG11 LMLAHGFVSSGLFITVTNLYDRFHTRIIRYYKGVVYTMPNIAFPISMNFV 2050
PG12 LMIAHGVVSPALFIAVTLLYERHHTRLVRYYRGVVMTMPNIAVPLSCNFV 2050
PG13 MMLAHGLVSSALFIAVTYLYERHHTRLIKYYRGITFSMPNMAIPLSCNFV 2050
PG14 MMLAHGFVSSALFIAITYLYERHHTRLIKYYRGVTLTMPNMGIPLSCNFV 2050
PG15 MMVSHGICSSGLFYLSYLFYARIWSRSFLLTRSMISLFPNMGLPPSLNFF 2050
PG16 MMIAHGLSSSGLFSMSNMMYEKSGSRSLFITKGFISLVPNMAAPPSINLL 2050
PG17 LMIAHGLISSALFCLANLSYERTFTRTLSLNRGLKLITPNLALPPTPNFS 2050
PG18 LMVAHGLVSSALFSLANTVYERSGTRTLAITRGLKLLLPNLGLPPSPNLI 2050

PG01 AEFLVFTGVMDINPFLAFIASLGLLLTATYSMWLYNRVCFGGVLGFILNN 2100
PG02 GEFLCLLAIFEVNTTVAILASLGIILSACYSLFLYNRVCFGGTLGIVVNR 2100
PG03 GEYLSILSSFNWNPIIGVFVCSGVILSAVYSFYFANRVTFGGVSGMMMNK 2100
PG04 GEYLSILSSFHWNPIIGVFVCTGVILSAVYSFYFANRVTFGGVSGIIINK 2100
PG05 GEYLSILSSFNWNPMVGVFVCSGVILSAVYSFYFANRITFGGVSGIIINK 2100
PG06 GEYLSILSSFNWNPIVGFFVCSGVILSAVYSFYFANRITFGGVSGIIINK 2100
PG07 GEFLSILAAFEYSFIVGALASLGMVLSAGYSIYLYNRVCFGGVLGIVLNR 2100
PG08 GEFFSLLAAFEYSYVVGVLASLGTVLSAGYSIYLYNRVCFGGGLGVVFNR 2100
PG09 GEFFSLLAAFEYSFIVGVLASLGMVLSAGYSIYLYNRVCFGGILGIVLNR 2100
PG10 GEFFSLLAAVEYNFGLGVLATSGMVWSAAYSLYLYNRISFGGGWGVVLNR 2100
PG11 GEFMSILSALQFSLLAILCPLAGMVLSAAYSLLFYNKVSFGGILGISINR 2100
PG12 GEFLSLVAAFSTSTSLGVLTSTSMVIAAAYSLYLYNRICFGGILGIILNR 2100
PG13 AEFFSLLAAFEYNIVIGVLAATGMVWSAAYSLYLYNRVSFGGVLGIVLNR 2100
PG14 GEFFSLLAAVEYNWGLGVLVTSGMVWSAAYSLYLYNRISFGGGWGVVLNR 2100
PG15 SEMYFFIGAFSLDWMVVGLSGILCFLSSCYCIYLYSSTSHGGLLIFSSSN 2100
PG16 SEAGLMISTVSISKFLMILLALMAFVSAVYTLFLFTNTQHGALMTICLQR 2100
PG17 GELLIVTNLISWNPWTALFLGLTALFTAIYSLYLYQSSQHSATLGLALNR 2100
PG18 GEILILSSLISWSVWLFPIVGFAQVFGAIYSLMIFQLSQQGGLMGILLNR 2100
```

```
PG01 RQLIFILLALELMIFFSLYLLVLAGAESAVALSLLVAFHRVRGDELTLSM  2150
PG02 KNIIVMLMSLELMLLFAFYILVVAAAESAIGLSILVAYYRVRGDSLTVTM  2150
PG03 NNMILMLMSIELMLLFSLMVLTVAAAESAIGLAILVAYFSLTGDTLSVTM  2150
PG04 NNIILILMSIELILLFSLMVLTVAAAESAIGLAILIAYFRLTGDTLSVTM  2150
PG05 NNIILILMSIELILLFSLMVLTVAAAESAIGLAILVAYYRLTGDTLSVTM  2150
PG06 NNIILILMSIELILLFSLMVLTVAAAESAIGLAILVAYYRLTGDTLSVTM  2150
PG07 SNLIIMLMSIELMLLFTIMILTVAAAESAIGLAILVAYYRIRGDSLTAIM  2150
PG08 SNLIIMLMSIELMLLFAIMIITVAAAESAIGLAVLVAYYRVRGDSLTSTM  2150
PG09 SNLIIMLMSIELMLLFTIMILTIAAAESAIGLAILVAYYRVRGDSLTSTM  2150
PG10 GHFIIMIISIELILLFTIMGLTIAAAESAIGLAIMVAYYRIRGDGLVAIM  2150
PG11 TNLILLLMSVELMLLFTIFIFTVAAAESAIGLAIIIAYFRLRGDSLSASM  2150
PG12 SNLIIMLMCVELVLLFAIMILTVAAAESAIGLAIMVNYYRLRGDVVTAVM  2150
PG13 GHLIIMLMSIELILLFTIMVLTVAAAESSIGLAIMVAYYRIKGDSLTAVM  2150
PG14 GHFIIMIVSIELILLFTIMGLTIAAAESAIGLAIMVAYYRIRGDSLVAIM  2150
PG15 KHLLVTLLSLEFLILNAFIFLSVTVCEGALGFSVLVSLVRSSGWLAKTII  2150
PG16 KHLLNTLLALEILMVMIFVLLTLAASEASIGLAMLVILIRAHGNWTTALF  2150
PG17 SHLLSALLCLELLLIASLLLITFSACEASAGLGLLVSISRTHGDIYSIVF  2150
PG18 LHFLSILLCLELLLIFNLFVLTLVACEASIGLSLMVGLSRTHSDQYFLVF  2150

PG01 MIPICFISFLVHIYSMGYMSDDPHLQRFFSYLSLFTFFMLIMVSSDNWLL  2200
PG02 LILVTTVSSLVHLYSISYMDGDPHLPRFMSYLSLFTFSMVMLVTADSYLL  2200
PG03 LLVISSISTLVHIYSTSYMSDDPHVPRFLCYLSLFTFLMMVLVTSDNYLQ  2200
PG04 LLVISSISTLVHIYSTSYMTNDPHVPRFLCYLSLFTFLMMVLVTSDNYLQ  2200
PG05 LLVISSISTLVHIYSTSYMSDDPHVPRFICYLSLFTFLMMVLVTSDNYLQ  2200
PG06 LLVISSISTLVHIYSTSYMSEDPHKTRFLCYLSLFTFLMMILVTSDNYLQ  2200
PG07 LMVITSVSALVHIYSTGYMSGDPHIPRFMSYLSLFTFLMIVLVTSDNYIQ  2200
PG08 LILITSVSALVHIYSTGYMSEDPHIPRFMSYLSFFTFLMIVLVTSDNYVQ  2200
PG09 LIVITSVSALVHIYSIGYMSGDPHIPRFMSYLSLFTFLMIVLVTSDNYIQ  2200
PG10 LLVITTISTLVHIFSTAYMLGDPHIPRFMSYLSFFTFLMVVLVSSDNYLQ  2200
PG11 ILAVNIVSFLVHMYSVSYMKGDPHTSRFMGYLSLFTFFMLILVSSQNYLQ  2200
PG12 LIVVTTISGLVHVYSTSYMREDPHLPRFMSYLSLFTFFMLVLVTSNNYVQ  2200
PG13 LIVVTSISTLVHIFSTAYMDGDPHVPRFMSYLSLFTFFMILLVTSDNYPQ  2200
PG14 LLVITTISTLVHIFSMAYMQGDPHIPRFMSYLSLFTFLMMVLVTSDNYPQ  2200
PG15 HLLSSLNSSQVMIYSSYYMMGETFVNRFMYMMLGFISSMVLLIMSSDGLS  2200
PG16 SAIVCYISASVMIFTKNYMSQDIFIKRFTWIVMLFVLSMNLLIFIPSLAT  2200
PG17 TSIALFITYNIVVFSSYYMAHDSKINTFTQQLTIFLLAMIILVSSNNIFQ  2200
PG18 LSVALIVTWSIMEFSFYYMTEDPNSSAFFRLLTIFLLNMLILTCSNSLFL  2200

PG01 LFLGWEGVGLVSYLLIGFWFTIKANQSALKAFLMNRIGDTGLFLVIAKSG  2250
PG02 CVWGWESVGICSYLLINFWYTIQANKSAIKAMVMNRVGDMTLFLAVGKSA  2250
PG03 LFIGWEGVGVCSYLLVAFWTTIQANKAAIKAIVVNRVGDVGVILCIGKSA  2250
PG04 LFIGWEGVGVCSYLLVAFWTTIQANKAAIKAIVVNRVGDVGVILCIGKSA  2250
PG05 LFIGWEGVGVCSYLLVAFWTTIQANKAAIKAIVVNRVGDVGVILCIGKSA  2250
PG06 LFIGWEGVGICSYLLVAFWTTIQANKAAIKAIIVNRIGDVGVILCIGKSA  2250
PG07 LFIGWEGVGLCSYLLINFWLTIMANKAAIKAMLINRVGDIGLVLAVGKSA  2250
PG08 LFIGWEGVGLCSYLLINFWLTIKANKAAMKAMLINRVGDIGLLLAVGKSA  2250
PG09 LFIGWEGVGLCSYLLINFWLTIKANKAAIKAMLINRVGDIGLVLAVGKSA  2250
PG10 LFIGWEGVGLCSYLLINFWLTVEANKAAIKAMLVNRVGDIGLILAVGKSA  2250
PG11 LFIGWEGVGLCSYLLINFWYSIQANKAAIKAMLVNRLGDAALIAAIGKSA  2250
PG12 LFIGWEGVGLCSYLLINFWFTIQANKAAIKAMLINRIGDIGLMLAVGKSA  2250
PG13 LFIGWEGVGLCSYLLINFWLTIEANKAAIKAMLVNRVGDIGFVLAVGKSA  2250
PG14 LFIGWEGVGLCSYLLINFWWTVEANRAAIKAMLVNRVGDIGLILVVGKSA  2250
PG15 LMLGWDGLGITSYLLIMFYKNYNSSSSGMITILSNRVGDVLILWSFTKSA  2250
PG16 LLLGWDGLGLVSFCLVIYYQNYKSLGAGLMTAFMNRIGDVAILLGMTKSA  2250
PG17 LLIGWEGVGFLSFLLIGWWSTSDANTAALQAIIYNRFGDIGILLATGKSA  2250
PG18 IFLGWEGGGFLSFLLISWWTTNDASSSALEAVITNRIGNIGLITAAGKSA  2250
```

```
PG01 QFGLHAWLPDAMEGTPVSALIHAATMVTAGIFLLLRFSPLFGSIFLVWLG 2300
PG02 QLGLHTWLPDAMEGTPVSALIHAATMVTAGVFLLIRSSPIIELVIITIVG 2300
PG03 QLGLHTWLPDAMEGTDSNGSRAPQHKWTAGVFLMIRSFPLFELLIITIFG 2300
PG04 QLGLHTWLPDAMEGTPVSALIHAATMVTAGVFLMIRSFPLFELLIITIFG 2300
PG05 QLGLHTWLPDAMEGTPVSALIHAATMVTAGVFLMIRSFPLFELLIITIFG 2300
PG06 QLGLHTWLPDAMEGTPVSALIHAATMVTAGVFLMIRSFPLFELLIITIFG 2300
PG07 QLGLHTWLPDAMEGTPVSALIHAATMVTAGVFLIIRSGPLFELTVVTILG 2300
PG08 QLGLHTWLPDAMEGTPVSALIHAATMVTAGVFLIIRSGPLFELTIVTSLG 2300
PG09 QLGLHTWLPDAMEGTPVSALIHAATMVTAGVFLIIRSGPLFELIIVTILG 2300
PG10 QLGLHTWLPDAMEGTPVSALIHAATMVTAGVFLLIRSSPFFELMIVTIVG 2300
PG11 QIGLHTWLPDAMEGTPVSALIHAATMVTAGVYLIIRSSPLFELIVTALVG 2300
PG12 QLGLHTWLPDAMEGTPVSALIHAATMVTAGVFLIIRSAPLFDTIIVGLVG 2300
PG13 QLGLHTWLPDAMEGTPVSALIHAATMVTAGVFLLIRSSPLLELMVVTIVG 2300
PG14 QLGLHTWLPDAMEGTPVSALIHAATMVTAGVFLLIRSSPLFELVVVTILG 2300
PG15 QLPFSAWLPAAMAATPVSSLVHSSTLVTAGIYLMIRLSPSFECFLLVVMG 2300
PG16 QLPFCSWLPAAMAATPVSALVHSSTLVTAGVFLLIQFYPFLSSTSLMIIS 2300
PG17 QFGLHPWLPAAMEGTPVSALLHSSTMVVAGIFLLIRVSPIISIQAALLLG 2300
PG18 QFGLHPWLPALLEGTPVSALLHSSTMVVAGVFLLVRTSELFSHSLVLILG 2300

PG01 GITALFGAVYGYVQTDMKRTIAYSTTSQLGYMVLACGLAHLMNHAFFKAL 2350
PG02 TLTALFAASIGLVQNDIKKVIAYSTCSQLGYMVLACGLFHLFTHGFFKAL 2350
PG03 ALTAFFAATVGLVQNDLKKVIAYSTCSQLGYMVLIIGLFHLFNHAFFKAL 2350
PG04 ALTAFFAATVGLVQNDLKKVIAYSTCSQLGYMVLIIGLFHLVNHAFFKAL 2350
PG05 ALTAFFAATVGLVQNDLKKVIAYSTCSQLGYMVLIIGLFHLVNHAFFKAL 2350
PG06 ALTAFFAATVGLVQNDLKKVIAYSTCSQLGYMVLIIGLFHLVNHAFFKAL 2350
PG07 ALTAFFAATVGVVQNDLKKVIAYSTCSQLGYMTMVCGLFHLMNHAFFKAL 2350
PG08 ALTAFFAATTGVVQNDLKKVIAYSTCSQLGYMVMACGLFHLLNHGFFKAL 2350
PG09 ALTAFFAATTGVVQNDLKKVIAYSTCSQLGYMVMICGLFHLVNHAFFKAL 2350
PG10 SLTVLFAATVGVIQNDLKKVIAYSTCSQLGYMVVACGLFHLMNHAFFKAL 2350
PG11 SITALFAATIGLAQNDIKKIIAYSTCSQLGFMVLSCGLFHLVNHAFFKAL 2350
PG12 SLTAFFAATVGLVQSDIKKVIAYSTCSQLGYMVMACGLYHLLTHAFFKAL 2350
PG13 SLTAFMAATIGLVQNDLKKVIAYSTCSQLGYMVMACGLFHLMNHAFFKAL 2350
PG14 SLTALIAATIGVVQDDLKKVVAYSTCSQLGFMVVACGLFHLMNHAFFKAL 2350
PG15 ALTSFFSGLAAFGENDLKRVIALSTLSQLGVMMFSLGYFHLFAHALFKAL 2350
PG16 SMTMVMAGISANFETDLKKIIALSTLSQLGVMMLSVSLFHLFTHAMFKAL 2350
PG17 SITAFFAASCAITQHDLKKIIAFSTTSQLGLMMVAIGFFHICTHGFFKAM 2350
PG18 GTTALFAASTAIAQHDIKKIIAYSTTSQLGLMVTAIGFFHICTHAFFKAM 2350

PG01 LFLSAGVVIHAVHDQDVRRMGGLIRLMPITYVTTLIGSLSLVLPFLTGFY 2400
PG02 LFLGAGSVIHAMGDQDMRKLGGLIKRIPYTYSMMLIGSLSLMFPFLAGFY 2400
PG03 LFLSAGAVVHSTNYQDMRKMGGLIHSIPFTYTMMLIGSFSIMLPYLTGFY 2400
PG04 LFLSAGAVVHSTNTQDMRKMGGLIHSIPFTYTMMLIGSFSIMLPYLTGFY 2400
PG05 LFLSAGSVIHSMTIQDMRKMGGLIHSIPFTYTMMLIGSFSIMLPYLTGFY 2400
PG06 LFLSAGSVIHCLTVQDMRKMGGLINTIPFTYTMMLIGSFSIMLPYLTGFY 2400
PG07 LFLSAGSVIHAVSDQDMRKMGGLIKSIPLTYTMILIGSLSLMFPYLTGFY 2400
PG08 LFLSAGSVIHAVNGQDMRKMGGLIRSIPFSYAMILIGSLSLMFPYLTGFY 2400
PG09 LFLSAGSVIHAVSDQDMRKMGGLIKSIPLTYTMILIGSLSLMFPYLTGFY 2400
PG10 LFLSAGSVIHALSDQDIRKMGGLIKLIPLTYIMVVVGSLSLMFPYLTGFY 2400
PG11 LFLGAGSIIHSMLDQDIRKMGGINITQPISYVFIVIGSLSLAFPFMAGYY 2400
PG12 LFLGAGSIIHALLDQDLRKMGGIIRGLPLTYILMLIGSLSLAFPYLSGFY 2400
PG13 LFLSAGSVIHALADQDMRKMGGLIRSIPFTYTMIVIGSLSLMFPYLTGFY 2400
PG14 LFLSAGSVIHVLFDQDTRKMGGLGRSIPLTYIMVVVGSWSLMFPYLTGFY 2400
PG15 LFMCSGVVIHSLGVQDNRRMGGVSSMLPYTSYIILVCSLSLMFPYLSGFF 2400
PG16 LFLCAGNIIHSHNNQDIRKMSHLWIQMPFSSTCFNIANLALCFPFMAGFY 2400
PG17 LFLCSGSIIHNSLDQDLRKGGGLAFSLPITSACLTLGSMALAVPFLAGFY 2400
PG18 LFLCSGSVIHSLSDQDLRKMGGLSKLLPVTSSCLILGSLALMAPLLAGFY 2400
```

```
PG01 SKDYILQMGLAAAYFTTFYSLKLLHRVFTITLTIASIFWGSHLPWWIDFV  2450
PG02 SKDAILELGTLAAGMTAYYSIRLIYMTFLFILSIGSIFFGKWIPVIISIS  2450
PG03 SKDLILELGTFAAFLTAFYSIRLVYITFLSLLCFGAIFFGKVLPTLAGVF  2450
PG04 SKDLILELGTFAAFLTAFYSIRLVYITFLSLLCFGAIFFGKVSPTLAGVF  2450
PG05 SKDLILELGTVAALLTAFYSIRLVYITFLSLLCFGAIFVGKVMPTFMGLF  2450
PG06 SKDLILELGTVAALLTAFYSIRLVYITFLSLLCLGAIFVGKIMPTLMGLF  2450
PG07 SKDLILELGSFSALLTAFYSIRLIYLTFLLLLAIGSILVGKITPLFLSLL  2450
PG08 SKDLILELGSFSALLTAFYSVRLIYLTFLLLLGFGSIFVGKITPFVLSLL  2450
PG09 SKDLILELGVFSALLTAFYSIRLIYLTFLLLLAFGSIFVGKVVPLLLSLL  2450
PG10 SKDLILELGGFSALLTTLYSIRLVYLTFLIILALGSLFVGKLLPVFLSLG  2450
PG11 SKDLLLELGIFATLLTALYSFKLILRSFLLVLAMGSITSGKLLPLLVTIA  2450
PG12 SKDLILELASITAFLTAFYSFRLIYLSFLLVLGVGSILIGSLAPVIMSVT  2450
PG13 SKDLILELGVFSALLTAAYSLRLIYLTFLILLALGSIFVGKLMPVIFSLF  2450
PG14 SKDLILELVDVAAFLTTVYSIRLVHLTFLVLLALGSLFVGKLMPVFFSLG  2450
PG15 SKDLIIELMLVSCLLTSTYSSRIAMVCLLFVLYWGAVMGGSFLLLSLITV  2450
PG16 SKDVIIENMFLATILTASYSARLSLFIFLTMLAAGAITSGKLMATFATLT  2450
PG17 SKDLILELAIIATTLTATYSSRIILASFIKNLAFGATLVGKTLTLLIAFI  2450
PG18 SKDLILELSIVATMLTAVYSFRIIFFCFLLRLATGTIASGKGTPLIVPII  2450

PG01 PDNGFLAWFGPHGAVYFLNSFAARLDLLSSRFIPHLSVLFLCGLYLAGGG  2500
PG02 GDRGWIEIFGPFGISKSISYITKNFSALQSGFIYNYSVLGLVMCFANSSI  2500
PG03 GDRGWLELFGPQGAGYLGTRVSQAISNLQSGVISRYSVFWLILAFIGSSG  2500
PG04 GDRGWLELFGPQGAGSLGTRVSQAISNLQSGVISRYSVFWLILAFIGSSG  2500
PG05 GDRGWLELFGPQGISTIALRVSQFISNLQSGVISRYSVFWLILTFIGSSG  2500
PG06 GDRGWLELFGPQGISTIALRGTKFISNLQSGVISRYSVFWLILTFIGSSG  2500
PG07 GDRGVLEIIGPRGISKILIKLTQNASNLQSGVVFNYSVLWLIVAFTSATA  2500
PG08 GDRGLLEIIGPKGAPQLLIRSTQRVSNLQSGTVFNYSVLWLVVAFIGASA  2500
PG09 GDRGILEIIGPKGISKVLIKLTQVISNFQSGMVFNYSILWLIVAFSSAAV  2500
PG10 GDKGTLELVGPKGVSNFLIGLTRGLSNLQAGQVFNYSIFWLIVVFIGSAV  2500
PG11 GDTGIIETFGPRGASIKIVKFSSFISSIQSGLLFNYSIIWLVLSFIGAAI  2500
PG12 GDRGILEILGPTGIAQFMVDRTKELSSLQSGLLFNYSVFWLVIAFVNAAV  2500
PG13 GDKGVLELIGPKGISQFMIRLTQEISNLQSGLVYNYSVFWLVVAFISSAA  2500
PG14 GDKGTLELVGPKGVSNFLVKLTQGLSNLQSGLVFNYSIFWLIVVFVGSAV  2500
PG15 GDCGWVEEAGPSLIHHNSLRGSSLFSFLTSSPYKVLFTLSLFVQTLLICV  2500
PG16 GDLGWMETIGGEGLLNKIMTLTTINQKNQSNLFNLFLGLLLLFMSLFISI  2500
PG17 GDQGWGEQIGAQGIGISNTTLAQKTQFLQSGLIKQYSALGLVLATCGLSG  2500
PG18 GDRGWQENIGPQGIAPTSTALSKISQAGQIGLIKRYSALGLVVFSVPGSF  2500

PG01 IFLLLDFLGLTLIIVYVGAIAILFLFTIFHAYPLALILVGFLLLVVMVAI  2550
PG02 LLLMLGFLALLFLIVYVGALAILFLFVLYTHGYIYFIVSSIVLLIAMVGA  2550
PG03 LFIVLGFLGLIFLIVYVGAICIIFLFVLYINYYYVFILVSFILLVAMIGA  2550
PG04 LFIVLGFLGLIFLIVYVGAICIIFLFVLYINYYYAFILVSFILLVAMIGA  2550
PG05 LFIVLGFLGLIFLIVYVGAICIIFLFVLYINYYYAFILVSFILLVAMIGA  2550
PG06 LFIVLGFLGLIFLIVYVGAICIIFLFVLYINYYYAFILVSFILLVAMIGA  2550
PG07 LFILLEFIALMFLIVYVGAIAILFLFVLYTNYYYLFIIASFILLVAMIGV  2550
PG08 LFILLEFLALILLIVYVGAIAILFLFVLYTNYCYLFILASLILLVALIGA  2550
PG09 LFIFLEFIAFIFLIVYVGAIAILFLFVLYTDYYYYFLLASFVLLVAMIGA  2550
PG10 FFLWLGFVALMFLIIYVGAIAILFLFVLYIACYYLFILASFILLVAMVGA  2550
PG11 VFIQLQFIALATLIIYVGAIAILFIFVLYNSYAPHLIVASLILLVAMIGA  2550
PG12 MFISLGYIGLIFIIVYVGAIAILFLFVLYTTYYELVLIASLVLLVAMIGA  2550
PG13 LFILLGFIALMFIIIYVGAIAILFLFVLYTDCYYLFILVSFILLVAMLGA  2550
PG14 LFLLLGFVALMFLIIYVGAIAILFLFVLYIACYYLFILASLILLVAMIGG  2550
PG15 MLKNVSWISLILFLIFLGGILVMFIYVVNSLTMLAYSFMVVYLFLALLLV  2550
PG16 LIFFGSWFSFILFLIYIGGLLVMFAYVFSPFNLLIIVSLALILFFVLICV  2550
PG17 VLMLGGFVGLILFLMYLGGMLVVFAYSLYLDSWAGLLLGAFGLFVCLFVV  2550
PG18 VLSFLGFVPIVLFLVYIGGMLVVFPYSFYNSGGVLVILGVFVLLVALVGA  2550
```

PG01 IKI 2553LAAAYFTTFYSLKLLHRVFTITLTIASIFWGSHLPWWIDFV 2450
PG02 IVL 2553TLAAGMTAYYSIRLIYMTFLFILSIGSIFFGKWIPVIISIS 2450
PG03 IVL 2553TFAAFLTAFYSIRLVYITFLSLLCFGAIFFGKVLPTLAGVF 2450
PG04 IVL 2553TFAAFLTAFYSIRLVYITFLSLLCFGAIFFGKVSPTLAGVF 2450
PG05 IVL 2553TVAALLTAFYSIRLVYITFLSLLCFGAIFVGKVMPTFMGLF 2450
PG06 IVL 2553TVAALLTAFYSIRLVYITFLSLLCLGAIFVGKIMPTLMGLF 2450
PG07 IVL 2553SFSALLTAFYSIRLIYLTFLLLLAIGSILVGKITPLFLSLL 2450
PG08 IVL 2553SFSALLTAFYSVRLIYLTFLLLLGFGSIFVGKITPFVLSLL 2450
PG09 IVL 2553VFSALLTAFYSIRLIYLTFLLLLAFGSIFVGKVVPLLLSLL 2450
PG10 IVL 2553GFSALLTTLYSIRLVYLTFLIILALGSLFVGKLLPVFLSLG 2450
PG11 IIL 2553IFATLLTALYSFKLILRSFLLVLAMGSITSGKLLPLLVTIA 2450
PG12 ILL 2553SITAFLTAFYSFRLIYLSFLLVLGVGSILIGSLAPVIMSVT 2450
PG13 IVL 2553VFSALLTAAYSLRLIYLTFLILLALGSIFVGKLMPVIFSLF 2450
PG14 IVL 2553DVAAFLTTVYSIRLVHLTFLVLLALGSLFVGKLMPVFFSLG 2450
PG15 IDF 2553LVSCLLTSTYSSRIAMVCLLFVLYWGAVMGGSFLLLSLITV 2450
PG16 VKI 2553FLATILTASYSARLSLFIFLTMLAAGAITSGKLMATFATLT 2450
PG17 LCL 2553IIATTLTATYSSRIILASFIKNLAFGATLVGKTLTLLIAFI 2450
PG18 LII 2553IVATMLTAVYSFRIIFFCFLLRLATGTIASGKGTPLIVPII 2450

PG01 PDNGFLAWFGPHGAVYFLNSFAARLDLLSSRFIPHLSVLFLCGLYLAGGG 2500
PG02 GDRGWIEIFGPFGISKSISYITKNFSALQSGFIYNYSVLGLVMCFANSSI 2500
PG03 GDRGWLELFGPQGAGYLGTRVSQAISNLQSGVISRYSVFWLILAFIGSSG 2500
PG04 GDRGWLELFGPQGAGSLGTRVSQAISNLQSGVISRYSVFWLILAFIGSSG 2500
PG05 GDRGWLELFGPQGISTIALRVSQFISNLQSGVISRYSVFWLILTFIGSSG 2500
PG06 GDRGWLELFGPQGISTIALRGTKFISNLQSGVISRYSVFWLILTFIGSSG 2500
PG07 GDRGVLEIIGPRGISKILIKLTQNASNLQSGVVFNYSVLWLIVAFTSATA 2500
PG08 GDRGLLEIIGPKGAPQLLIRSTQRVSNLQSGTVFNYSVLWLVVAFIGASA 2500
PG09 GDRGILEIIGPKGISKVLIKLTQVISNFQSGMVFNYSILWLIVAFSSAAV 2500
PG10 GDKGTLELVGPKGVSNFLIGLTRGLSNLQAGQVFNYSIFWLIVVFIGSAV 2500
PG11 GDTGIIETFGPRGASIKIVKFSSFISSIQSGLLFNYSIIWLVLSFIGAAI 2500
PG12 GDRGILEILGPTGIAQFMVDRTKELSSLQSGLLFNYSVFWLVIAFVNAAV 2500
PG13 GDKGVLELIGPKGISQFMIRLTQEISNLQSGLVYNYSVFWLVVAFISSAA 2500
PG14 GDKGTLELVGPKGVSNFLVKLTQGLSNLQSGLVFNYSIFWLIVVFVGSAV 2500
PG15 GDCGWVEEAGPSLIHHNSLRGSSLFSFLTSSPYKVLFTLSLFVQTLLICV 2500
PG16 GDLGWMETIGGEGLLNKIMTLTTINQKNQSNLFNLFLGLLLLFMSLFISI 2500
PG17 GDQGWGEQIGAQGIGISNTTLAQKTQFLQSGLIKQYSALGLVLATCGLSG 2500
PG18 GDRGWQENIGPQGIAPTSTALSKISQAGQIGLIKRYSALGLVVFSVPGSF 2500

PG01 IFLLLDFLGLTLIIVYVGAIAILFLFTIFHAYPLALILVGFLLLVVMVAI 2550
PG02 LLLMLGFLALLFLIVYVGALAILFLFVLYTHGYIYFIVSSIVLLIAMVGA 2550
PG03 LFIVLGFLGLIFLIVYVGAICIIFLFVLYINYYYVFILVSFILLVAMIGA 2550
PG04 LFIVLGFLGLIFLIVYVGAICIIFLFVLYINYYYAFILVSFILLVAMIGA 2550
PG05 LFIVLGFLGLIFLIVYVGAICIIFLFVLYINYYYAFILVSFILLVAMIGA 2550
PG06 LFIVLGFLGLIFLIVYVGAICIIFLFVLYINYYYAFILVSFILLVAMIGA 2550
PG07 LFILLEFIALMFLIVYVGAIAILFLFVLYTNYYYLFIIASFILLVAMIGV 2550
PG08 LFILLEFLALILLIVYVGAIAILFLFVLYTNYCYLFILASLILLVALIGA 2550
PG09 LFIFLEFIAFIFLIVYVGAIAILFLFVLYTDYYYYFLLASFVLLVAMIGA 2550
PG10 FFLWLGFVALMFLIIYVGAIAILFLFVLYIACYYLFILASFILLVAMVGA 2550
PG11 VFIQLQFIALATLIIYVGAIAILFIFVLYNSYAPHLIVASLILLVAMIGA 2550
PG12 MFISLGYIGLIFIIVYVGAIAILFLFVLYTTYYELVLIASLVLLVAMIGA 2550
PG13 LFILLGFIALMFIIIYVGAIAILFLFVLYTDCYYLFILVSFILLVAMLGA 2550
PG14 LFLLLGFVALMFLIIYVGAIAILFLFVLYIACYYLFILASLILLVAMIGG 2550
PG15 MLKNVSWISLILFLIFLGGILVMFIYVVNSLTMLAYSFMVVYLFLALLLV 2550
PG16 LIFFGSWFSFILFLIYIGGLLVMFAYVFSPFNLLIIVSLALILFFVLICV 2550
PG17 VLMLGGFVGLILFLMYLGGMLVVFAYSLYLDSWAGLLLGAFGLFVCLFVV 2550
PG18 VLSFLGFVPIVLFLVYIGGMLVVFPYSFYNSGGVLVILGVFVLLVALVGA 2550
