# Supplementary material for: Comparative Genomics of Large Mitochondria in Placozoans
Source: PLoS Genet. 2007 Jan 12;3(1):e13. doi: 10.1371/journal.pgen.0030013 (PMC1781491; doi:10.1371/journal.pgen.0030013)
Supplement: Dataset S2 — (68 KB PDF) [file pgen.0030013.sd002.pdf]

Dataset S2: Amino acid sequence alignment of *RVT-IM*

Three of the four placozoan mtDNAs sequenced contained an open reading frame encoding a reverse transcriptase and an intron group II maturase domains (*RVT-IM*). Below is the amino acid sequence alignment for this gene. Box shade ■ indicates identical amino acids and box shade ■ indicates chemically similar, but not all identical amino acids.

**Taxon name**	**Abbreviation**
BZ2423	B1
Trichoplax	B2
BZ49	A1

```
B1 -----------------------------------------------MNR    3
B2 MLLT----DRR-----ATVRLLKEDGE---TRPPVSEQLADKVGLTYHNI   38
A1 MYVVGMDVDTRAYFTAATMIIAVPTGIKIFVRQPIVRMNGQLMISNCHNV   50

B1 LIV------------N----P---RS-----FW----RVLSADRFNR---   22
B2 LIVFKHYILGKKHVRNGIIGPSTDRSGAIGADWSSGPRFISTTSSLKDE-   87
A1 RTFSELGSSERK-LKN--IAS---RK----KAW--MERNVEVKGLQNVLS   88

B1 ---IQCV-YIIFK----P-GSN-----P---KNRAPGPPKK----RNSLF   51
B2 --QVNCKPEVFFGGFCLPIGAEGALGRPG--ALRAPGAPFNSKLAQRALL  133
A1 MVRANLWSSLLYG---KSLGRNGAFMLPMTIWILVFGVVQHKTGGGEALW  135

B1 --LGS----------RFSLIFDCFY--IGIHKLR--ANQGIQWIRDQ---   82
B2 RALGGPPLRGPNRAPRFSLICDCFY--IGIHKLR--ADQGIHWIRDR---  176
A1 APLPPQAER------RPKDGYKHTYGSLGLSKEINLYDNRVRIVRKRGAF  179

B1 --KGIFQTREIQCFLVPFSIPVNHLFPP-QLG-Q-----DSW--------  115
B2 --KGIFQTREIQCFLVPFSIPVNHLFPPISIGPQRIPKGSFWGRS-----  219
A1 GAPGFFS-KDWQPSFFPTKWPYKICWGPGSNG-SVVSFFRYYVAEPNSIK  227

B1 ------LPG-------WDRGSVVRYRKGPN-----FQIKLFFR-------  140
B2 ------APSAPILFCRWGRGSVVRYRKGPN-----FQIKLFFRRDLEGPK  258
A1 NQAKRGAPAP---LAPWPRKAAPLAP-GPGREKVIKKIRDLTKRVLKDPG  273
```

```
B1  --------AAGCSVVTLTLSREA-------------P--QKK--GIPF--  163
B2  -------TTLGPAPKALAPSRAGSNGLRSFGAWPMVPGTQKM--GFPLFL  299
A1  RVIDRKLYNLVCNPFMLEFAYNNIK--KKGG-----KIDLKKGGGNPV--  314

B1  --SVGLYRLYSSKRRLRRPVLKLKTLWNG----NKKNK----NFIVKDLF  203
B2  WGAVGLYRLYSSKRRLPRPVLKLRTLWNG----SKKNK----NFIVKDLF  341
A1  --GSPSGSLLTTKGIVPKTLDEIS--WNILLALAKELKEEKFQFKPKKGS  360

B1  RYIMDK---DLWFLATKEYPK-KNILVLQKIVRIILESIYEPLFSFHSFG  249
B2  RYVMDK---DLWFLATDRDPK-KNIV--RQIVRIILESIYEPLFSFHSFG  385
A1  KIIGSQRVSSIPFTIAPSDPLGPKIV--LEVMRMLLNIIFEPSFLDCSHG  408

B1  FRPGKTQHTAIKKIREN---FKGVNWFIEGKGNKKLCLMGASDRHQRSP-  295
B2  FRPGKTQHTTIKKIREN---FKGVNWFIEGKGS--LKSFGDTP-PQRGPF  429
A1  YRSGRGLHSVFNYLKTGALCTPGVR-IIE--GN--KCFFKIDH-------  446

B1  -DPLLNLLKKKIKDKKFISFLLSNIF---VHTSP-KTMGPAPVVRSVRPG  340
B2  GAPLLNLLKKKIKDKRFISFLENNILE--TPETPFKTMRPDGSVGSARPG  477
A1  -SILMNIIEKKILDRQFTKLLWKSLGAGYIEGEGGIRRFPPNLAAPLAPG  495

B1  LLIEPLLFNIIFHEFDMYMEKILKNLGS-TGSWGRVP-RPGGAKHPKSKL  388
B2  FILEPLLFNIIFHEFDMYMEKLIRNIK--KGGRKKAPLQPLGALK-KSKL  524
A1  SIISPILYNIYLHKLDEYILKLMVSFDIGKGAKSRDPFDPAYAPR----F  541

B1  GVLIRESQPARRLLATRTRSSYGRRRSKNYLIIKHLGYVRYA----TFFL  434
B2  -VFEGTKGPTDK--NNRADPTDPSRVPCGGPIIKHLGYVRFAGSNGSPFL  571
A1  -LYSGGGSPEGS---PPPKKKKG-LVPGGGEPIGLSPGPKGS--------  578

B1  IGILGPRSAICKLKKQICADRHQWCPGAALEPFNLVISSSDPKRNQLPR-  483
B2  IGIRGPRSAIFKLKEQICRLGGVGRGYGAAQPQ--KIGSVGAHWIGAPKD  619
A1  A-SLGALSAPGSVGSALCWADFPYKRISYFRLFNYWIIAFKGSYSETVEI  627

B1  ---FLGYIISQKIRKKYKRGGGSVYRRSAEWRLTP--KGEGHSNAFESP-  527
B2  T--FWG------PRRPTK-GLLSVN-RSLDP-LDPNWSSWGHNIVISSSD  658
A1  HSKVLG-----FCRDVLKLKGGNIRCPSVVNDLDV------KDAPFVG--  664
```

```
B1 PLTN-HW----IRGPGTEPRRLPALSAPGQLSSYRMYLKMDSNIVITYLA  572
B2 PKRNQQR----FLGYIISQKATNKYKRGGGSNGSRMYFKMDNNIVITYLA  704
A1 PIKNLQKDKIVIFGINVFRSKHVKFSRKGEPCGLRD-FSINLSSIKKKLR  713

B1 KKGFWDGAPKGPIPK-GPFGPGSFNSIQGLWAQLERYYHLANDKQQKLGR  621
B2 KKGFCDGGG-APIPNFGAEGAGSLNSVQGLWALFSRYYHLANDKQQKLGR  753
A1 EINILKGEK--PIPR-------------LAWSPLG--------SEQ----  736

B1 IGYIFEHSFAKYFAACFRLKTRRQVFKKIGWSYDGPPFKPLFFGFRVPV-  670
B2 ISYILKHSIAKYFAACFRLKTRRQVFKKIGGRS-LTLFKPLSFGFRAPVG  802
A1 ILLLFNSVLKEYFGHFDLVRGR-------GAK-----------GSGAPQK  768

B1 -VPAKVAR-------QRSHSF--FCHNVIKYPPIN-FNFFINPPNLCTR-  708
B2 GTPRKGPQPVGAFGAQRSHSWGAFGADVKKYPSINSTNFFIKSPDIDSKS  852
A1 ADPMDPGR--------------------LRRPHFARFKFWLK--------  790

B1 IGIVEGISVFLTNTGVFQIQTT-------FLIDCA---QDIFLGPKG---  745
B2 LGIVEGISVSLTNTGVFQIQTTLGAEGAYFGVDSTTPQSTNSVGAEGAQF  902
A1 --WVITTSAERTLARKYNTSTN--------KIGGR---------PRG---  818

B1 ----------
B2 LHTFYTGRRL  912
A1 -------KR-  820
```
