# Supplementary material for: Comparative Genomics of Large Mitochondria in Placozoans
Source: PLoS Genet. 2007 Jan 12;3(1):e13. doi: 10.1371/journal.pgen.0030013 (PMC1781491; doi:10.1371/journal.pgen.0030013)
Supplement: Dataset S3 — (93 KB PDF) [file pgen.0030013.sd003.pdf]

Dataset S3: Amino acid sequence alignment of *polB*

Strain BZ49 contained an open reading frame similar to fungal DNA polymerase type B (*polB*). The following is an amino acid sequence alignment for this gene using BZ49 and five fungal taxa: *Agrocybe aegerita, Neurospora intermedia, Neurospora crassa, Hebeloma circinans,* and *Pleurotus ostreatus* with accession numbers: AAC33727, NP_049544, CAA39046, CAA72280, and NP_053000 respectively. Box shade ■ indicates that ≥ 60% of amino acids are identical and box shade ■ indicates chemically similar, but not all identical amino acids.

**Taxon name**	**Abbreviation**
BZ49	po1
Agrocybe aegerita	po2
Neurospora intermedia	po3
Neurospora crassa	po4
Hebeloma circinans	po5
Pleurotus ostreatus	po6

```
po1 --------------------------------------------------
po2 --------------------------------------------------
po3 --------------------------------------------------
po4 --------------------------------------------------
po5 --------------------------------------------------
po6 MSDDASAIAAGGGAASALIKSIPNASISTKAIGGTAVSIGVTAGVKIGVH     50

po1 --------------------------------------------------
po2 --------------------------------------------------
po3 --------------------------------------------------
po4 --------------------------------------------------
po5 --------------------------------------------------
po6 VGSKCIENDSMSKAIESLDESVKTRPYAEPTPDRIPSPDQNWINSPLEND    100
```

```
po1 --------------------------------------------------
po2 --------------------------------------------------
po3 --------------------------------------------------
po4 --------------------------------------------------
po5 --------------------------------------------------
po6 SIPLIDILGDLISLNLLEIFLLLCILLLLIIKNNLNKGSLNKMIWKVITK    150

po1 --------------------------------------------------
po2 --------------------------------------------------
po3 --------------------------------------------------
po4 --------------------------------------------------
po5 --------------------------------------------------
po6 FVPQIYQDKINNIIKTGKEYNNKINNIIIYVLILIIILLILGNLITCNEL    200

po1 --------------------------------------------------
po2 --------------------------------------------------
po3 -----------------------------------------MIKFYFTTF      9
po4 -----------------------------------------MIKFYFTTF      9
po5 -------------------------------------------SFQLILE      7
po6 YNNIDKYVLVYNYKINKKLLFLICVNKNLWSNNNNISLRKNINNVNLIGD    250

po1 --------------------------------------------------
po2 --------------------------------------------------
po3 HMKFPARLFSTSG---NRANIEQNSSNEVQNQIKYLKDFKKENP------     50
po4 HMKFPARLFSTSG---NRANFEENSSNEVQNQIKYLKDFKKENP------     50
po5 KIKSQEKRLKES----IRLVKDGVLRSDGKNKIGEGTDFFPSNI------     47
po6 CISKREYSLNQNSNTNNREININNLDKYHKEDLEKLYNFGLTNPDFVKNN    300

po1 --------------------------------------------------
po2 --------------------------------------------------
po3 -----------KLKWESTTEPKHMI-------------------------     64
po4 -----------KFNWESTTEPKHMI-------------------------     64
po5 -----------KRFMKTSLIAQVKG-------------------------     61
po6 IEFLNIMETLERRFDKSNDLTNDRIKQRKMKFNDGYIEVGEFKNIVLLTD    350

po1 --------------------------------------------------
po2 --------------------------------------------------
po3 -LSNIGEYEKND-------FNLKSLLNINEFNINVELLRQLFMKLDKNFT    106
po4 -LSNIGEYEKND-------FNLKSLLNINEFNINVELLRQLFMKLDKNFT    106
po5 -NNYGTLISKDE-------IEGANLNLIFNLNMETDSLYNLSLKNKR---    100
po6 DLSPIGDVQTIENEQDHILNESFNIYNMLEYKKRFEQILLNYCKEGKTYK    400
```

```
po1 --------------------------------------------------
po2 --------------------------------------------------
po3 YALGVILRNVDSNTN----ISVDRHFLVIFNTDPLIILQQIYDRIIFLSE   152
po4 YALGVILRNVDSNTN----ISVDRHFLVNFNTDPVIILQQIYDRIIFLSE   152
po5 YFVKVSLLNRDNEE------------------------------------   114
po6 MIFKVNLINVEISGIGEIQSKTTPSFLIHRHSNIDYILIKFSNSIMDLLF   450

po1 --------------------------------------------------
po2 --------------------------------------------------
po3 KYYLEPVDKLFIRLRKLNFKV---KNPVFHPIAKEKTNIHTTIPKGKSKL   199
po4 KYYLVPVDKLFIKLRKLNFKV---KNPVFHPIAKEKTNIHTTIPKGKSKL   199
po5 ----VLLLKVTIFLDKQSHDS---KNGLSFMNFQEMVNWTDSIKQG----   153
po6 QYNIADRVNINIFIKEWIDSSELKNIASLYPILNKKENSIVSKYLDKLNK   500

po1 --------------------------------------------------
po2 ----MVPSKTSP---------NRDTKIISMDLETILINNKHIPY----LL    33
po3 SRPEFVPHTMDLKFYGVETKHDKENNIRIFDNGNVILKVKIIKDGVNHLI   249
po4 SRAEFVPHTMDLKFYGVETKHDKEKNIRIFDNGKVILKVKIIKDGVYHLI   249
po5 AEGEVVERYSVETGYKITFKQDVITRVDKQFHSKTLVRSLRDYE-EDKNI   202
po6 NITDNVIDKTQLGLLGITRKSKSSLLVKGLNYGKIITNENLVSTYDKMIN   550

po1 --------------------------------------------------
po2 SWYDGN--------------------------------------------    39
po3 DVISRD-------NRLLYQF------------------------------   262
po4 DVISRD-------NRLLYQF------------------------------   262
po5 GSIDIE-------TYLNENN------------------------------   215
po6 NYYSLNDNDNDNYNRLNQDNKLLDIKYYKLSYGSIQSKKEYIIKVSNVDN   600

po1 --------------------------MYLTQD------------------     6
po2 ---------------ITKSYFIDSIENNIENN------------------    56
po3 --------EDVKHGDGLKRHWINDNMYYYYYDNT----------------   288
po4 --------EDVKHGDGLKRYWINDNMYYYYYDNT----------------   288
po5 --------EAVPYAIGFKTVKGTKLFYLDSYS------------------   239
po6 ITNEVSAYIEVKENNIIKLLKVEEWVDNIKYNQSEFTKNENTVIICIRNI   650

po1 -------------------------INPE-----Q---LIMFSIGRILIP    23
po2 --------------------------IEN---------MISRAMNDICIR    71
po3 -------------LVNVETPQKVGNIEPTKRDKRQDKKILAFDIETFQVP   325
po4 -------------LVNVETPQKVGNIEPAKRDKTQDKKILAFDIETFQVP   325
po5 -------------------------NPSN---------MILDCIQYMLVK   255
po6 KGSSQKMTFVNNQLENIEYKYNCKLLEESRLDLDKNLKIGTFDFETYLDK   700
```

```
po1 --------------------------------------------KYHNYT   29
po2 --------------------------------------------KYKNYK   77
po3 TGNGDSTMIAYACGFYDGNKSLTYYISDFISQREMLLACIKDMLKYDKHT  375
po4 TGNGDSTMIAYACGFYDGKKSLTYYISDFISQREMLLACIKDMLKYDKHT  375
po5 --------------------------------------------ENHNFK  261
po6 --NQNAIPYYIGCRTGDKKVFYKYSDYLNVDEMVLKFILDLMVIENDNRF  748

po1 IYIHNLKMFDVHFLIKAIGKT----DWKTNVIMD-DNNNIISLKLWKYN-   73
po2 VYLHNFAKFDGYFLVKYLSKLG---FIDNIIINK--GR-IITLKFIYNKY  121
po3 VYCHNFSKFDINFIIKILVQE----FVVEKIISK-DLD-ILSIKISYKFE  419
po4 VYCHNFSKFDINFIIKILVQE----FVVEKIISK-DLD-ILSIKISYKFE  419
po5 FYAHNMGEFDGVFLLKSLMTSSHLHDLKFNVYSNNDGK-IISLDIVKRIV  310
po6 YYAHNLSDFDGMFVLKSLINTSKSHDLKIKVLSKNDGT-IISLEIKKILI  797

po1 ------KSWIH-LKLKDSLLLLNTSLYNLGKQINTEYR-----------K  105
po2 S---------I--TFKDSYLLLPSSLRKLCKSFNTQTQ-----------K  149
po3 PKKKGGKAERHTITIADSCRLLPGSLDKLAKDHNIITK-----------K  458
po4 PKKKGGKAERHTITIADSCRLLPGSLDKLAKDHNIITK-----------K  458
po5 K-----QKKTIKITILDSYLLLPFSLKKVAKVFNCNES-----------K  344
po6 N------KKIIKITLLDSLHLLPFSLRDLGKVFSLKKNSFYNETKNIIGK  841

po1 DNFPYSFMTSE----TIYYKGVCPPDKYWNKPPKN-------------RV  138
po2 DIFPYLLDDIN-------YIGEVPDYKYFCNLEMEEYN---NYKS---NF  186
po3 GKFPYKFVNKD----NLEYVGLIPDYEYYIDPKKGEMITLFEWAA--MYT  502
po4 GKFPYKFVNKD----NLEYVGLLPDYEYYIDPKKGEMITLFEWAA--MYT  502
po5 GLFPYKFIRED----NINYKGVIPEIKYFTDLSIKDYN---KYSE--DLK  385
po6 GNFPHDFIQKLGIDKALEYKGLVPDIKYFDNLSEEDYITEIVNRIKNEEN  891

po1 QIWDARVETLKYLENDLKTLYEVINKYADEIFKKYQINITDHNTISSLSL  188
po2 KVWNFREEAIKYCNLDCISLYEILYKFNTLVFNKFELNINKYPTLPSLSF  236
po3 NKWSTRKETIIYLEKDIKALYQLMMEMSNNTYSTFRINITRVKTASALAF  552
po4 NKWSMRKETIIYLEKDIKALYQLMMEMSNNTYSTFRINITRVKTASALAF  552
po5 GIWDCKEQTIDYLVKDLDILYEIMHKFNDTIFREYHVNITRIRTISGLAF  435
po6 GVWDCEKELLKYLNGDIDTLYSVMYTFGEFIFDKFNINITRIRTYSGLAF  941

po1 KIFQSNF----------------------YDMSKN---T----LQTLTGI  209
po2 ALFKTKY-----------------------LKENE--------VHMLSGS  255
po3 LVYRTLFLPNEVEEEEENETTSPNNILSLFDKKEEKKLTPKYFLPKLKGR  602
po4 LVYRTIFLPNEVEEE--NETNSPNNILSLFDKKEEKKLTPKYFLPKLKGR  600
po5 LIYTAIY----------------------YKVKDKP-------IYYTSGK  456
po6 LIYTSRY----------------------YDSIKKP-------IFLTTGK  962
```

```
po1  SETQIRHAYRGGMVMVNKREIE---KGYLYDVNSLYPYAMLN-PMPMGHP   255
po2  IATNIRKSYTGGSVDMYIPLIEKDSKIFIYDINSLYPFSMKSFKFPIGNP   305
po3  LERAVRAAYFGGRNEIFIPIIN---NIFSFDFNSLYPTAMMM-PMPVGIP   648
po4  LERAVRAAYFGGRNEIFIPIIN---NIFSFDFNSLYPTAMMM-PMPVGIP   646
po5  LEQFIRKGYYGGIVDVLTEYTDF--ETYKYDVNSHYPCAMLK-PMPGGIP   503
po6  IDNYIRNAYYGGIVDSWTFYSEK--PLFKYDFKSHYPNQMRNNPMPGGLP  1010

po1  ELSN---DKELNNYFGFVYVEVSPP---NTNIPILPTP-IEYKEGQ----   294
po2  TFFKGDITRINKDAFGFFYCKIITP--EYLEHPILQTHLKTSDGIRTLAP   353
po3  VHTF---SKNLNEIFGFVRAKIITP---AINIPVLPCR-VKVNGVQKLIF   691
po4  VHTF---CKNLNEIFGFVRAKIITP---AINIPVLPCR-VKVNGVQKLIF   689
po5  AVST---EKNLDNIFGFVEAIVEAPTKEELRVAILPCK-KDGKTVLFR--   547
po6  IFST---ETNLDLIFGFVRAEVTAPSEKELRVAILPIKGPNGELITFR--  1055

po1  ---KFKGWYFSEELKNAQKYGYKIKLFGG-YKFKKQYKMFDTFVKTFYQM   340
po2  -LGSWEDMLFSEEMYNAMKYGYKFEILWG-YTFESK-NIFSEIISDLYKM   400
po3  PIGEWTGWYFSEELKLAVEYGYKIEVLES-YVFEKRDDPFKEYIEHFASI   740
po4  PIGEWTGWYFSEELKLAVEYGYKIEVLES-YVFEKRDDPFKEYIEHFASI   738
po5  --DTVEGVWWSEELKMAREYGYKILEIKTCIVFDKVEEQFDSYIKNIYSK   595
po6  --GTVEGTWFSEELKNAETYGYKIKVKDC-LQFEKVYNLFDDFVNEFYNL  1102

po1  KVEGDSTN----RMVAKTMLNSLYGRLGMK--EQYISAYFVNK-KDAEQV   383
po2  RLEYQKSDP--MNYIAKILMNSLYGRFGMDDNFTFSDIMDKDDYFNFEKL   448
po3  KDNTKGSK----KQMAKLLLNTLYGRTGMNDSAAEIKMLTTNE-LDNIQL   785
po4  KDNTKGSK----KQMAKLLLNTLYGRTGMNDSAAEIKMLTTNE-LDNIQL   783
po5  KLQAEKEKNEIQRLLNKLLMNSLYGRLGIKDNNNNLKIVKQDT-IKKGLE   644
po6  KTEAEMNKDDISRLIYKLILNSLSGRWGLRDLNTEMKVIESKD-LNFLNK  1151

po1  LKNKEAILEWEYDN-KMLIRT-------------QTRELSNKIKN-----   414
po2  DRNNSILDVTELNNNNFLVTTKNP-----------KVELDTILDN-----   482
po3  TNN--VIHEFEVDDDKHYVRYDKKPCPVLCAQSEKNYELLSYLDGE----   829
po4  TNN--VIHEFEVDDDKHYVRYDKKPCPVLCAQSEKNYELLSYLDGE----   827
po5  TEN--SDILSESNN-LYLVKSQGPLDPEILNIINKEKLFESHDKGFNAK-   690
po6  TEN--VDTLFESNR-LSLIKSHGPLDPEVVDLFSKENLIENKKINPFIED  1198

po1  --KLERPTPASIQIAAAITAYARIFM--SKYIQKSHYT--DTDSIVVSKP   458
po2  ---GSEKHDINIAIASAITAYSRIQMSKFKNNPNFKLFYSDTDSVYISKE   529
po3  --KDDGFIINSTSIAAATASWSRILM--YKHIINSAYT--DTDSIFVEKP   873
po4  --KDDGFIINSTSIAAATASWSRILM--YKHIINSAYT--DTDSIFVEKP   871
po5  --NPWKGVSSSVQLSAAITAYARMYLNKFKNIPGNEYLGGDTDSIILTHP   738
po6  KDKPWGKNKSAVQLSAAISAYGRISMSKVKNISDNLYFGGDTDSFILEKP  1248
```

```
po1 LPKREIGVSC-------------------------------ITVICVSSC   477
po2 LPEELVSNTELG------------------------------KMKKEGIC   549
po3 LDSAFIGEGCGKFKAEYNGQLIKRAIFISGKLYLLDFG-GKLEIKCKGIT   922
po4 LDSAFIGEGCGKFKAEYNGQLIKRAIFISGKLYLLDFG-GKLEIKCKGIT   920
po5 LEKEFVGKDLGLFKLEH---VIVEGFYLTKKFYMIITNKNEVIIKAKGIS   785
po6 LDSSMIGKKLGQAELEQ---VIVEAFFHCKKSYLIINDKNETIIKMKGIN  1295

po1 R-----------------FN----ILFPRRF-------------------   487
po2 DD---------------AVFLAPKVYGYKDINGKATD-------------   571
po3 KNKDNTTHNLDINDFEALYNGESRVLFQERWGRSLELGTVTVKYQKYNLI   972
po4 KNKDNTTHNLDINDFEALYNGESRVLFQERWGRSLELGTVTVKYQKYNLI   970
po5 NQNN----LLNYNTFLELFKGNTVTFPSLQFQKNYKTLEISIINS-----   826
po6 KPN----LKLNYNKFVQLFKGEDIKIKQLEFRKDYK--------------  1327

po1 --------------------LCS---------------------------   490
po2 --------------------------------------------------
po3 SGYDKREKLYSLGKWVNTSPLCINENFEVISKALVSDVGETWYRKRIHYN  1022
po4 SGYDKREKLYSLGKWVNTSPLCINENFEVISKALVSDVGESWYRKRIHYN  1020
po5 ---NKK--------------IKGITNPEVKYKIMN---RSIAYKTRFEYY   856
po6 -----------------------NN-EMLKFIIYIKQLKELKI-------  1346

po1 -------------
po2 -------------
po3 KYNHIIYDYLKVF  1035
po4 K------------  1021
po5 SL-----------   858
po6 -------------
```
